# Cobalt catalyzed sp^3^ C–H amination utilizing aryl azides†
†Electronic supplementary information (ESI) available. CCDC 1057198–1057200. For ESI and crystallographic data in CIF or other electronic format see DOI: 10.1039/c5sc01162k


**DOI:** 10.1039/c5sc01162k

**Published:** 2015-07-30

**Authors:** Omar Villanueva, Nina Mace Weldy, Simon B. Blakey, Cora E. MacBeth

**Affiliations:** a Department of Chemistry , Emory University , USA . Email: sblakey@emory.edu ; Email: cmacbet@emory.edu

## Abstract

A dinuclear Co(ii) complex supported by a modular, tunable redox-active ligand system is capable of selective C–H amination to form indolines from aryl azides in good yields at low (1 mol%) catalyst loading. The reaction is tolerant of medicinally relevant heterocycles, such as pyridine and indole, and can be used to form 5-, 6-, and 7-membered rings. The synthetic versatility obtained using low loadings of an earth abundant transition metal complex represents a significant advance in catalytic C–H amination technology.

## Abstract 2

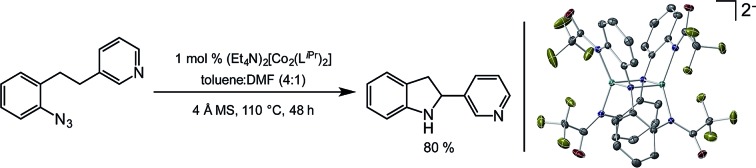
A dinuclear Co(ii) complex supported by a modular, tunable redox-active ligand system is capable of selective C–H amination to form indolines from aryl azides in good yields at low (1 mol%) catalyst loading.

## Introduction

The direct amination of C–H bonds in a mild, functional group tolerant manner represents an important technology for the synthesis of biologically active molecules, including natural products and pharmaceutical agents.^1^ Significant progress has been made in the development of catalysts capable of promoting metallonitrene initiated C–H amination utilizing carbamate,^2^–^6^ sulfamate,^2^,^7^–^12^ sulfonamide,^13^,^14^ sulfamide,^15^–^18^ and phosphoramide^19^ reagents for both intra- and intermolecular C–H amination, and in some cases, useful levels of enantio-induction can also be achieved.^20^–^24^

On occasion, these C–H amination reactions install the desired nitrogen functionality, but more frequently deprotection and subsequent elaboration are required. Robust C–H functionalization reactions to directly incorporate alkyl or aryl amines into a target molecule have proven to be more challenging and are the focus of this study.^25^

Alkyl and aryl azides have been recognized as important reagents for these reactions, offering the potential to deliver the C–H amination product in the presence of an appropriate catalyst, with nitrogen gas as the only byproduct. In early work, Driver and coworkers described [(cod)Ir(OMe)]_2_ as uniquely effective in catalyzing the intramolecular sp^3^ C–H insertion of a presumed metallonitrene intermediate obtained by aryl azide decomposition.^26^ Subsequent studies have shown that Rh_2_(esp)_2_ 27 and Fe(TPP)^28^ are also capable of inducing these reactions.

The pioneering studies of Tim Warren and coworkers established β-diketimide copper complexes as effective catalysts for C–H amination reactions with alkyl azides, but these reactions are complicated by competitive α-hydride migration.^29^,^30^

Recent reports by Betley and coworkers have established the advantage of incorporating a dipyrromethene redox-active ligand system^31^–^33^ at Fe(ii) centers to promote efficient catalytic C–H amination of aliphatic^34^ and olefinic^35^ substrates. Application of this ligand system in a Co(i) complex proved to stabilize a cobalt(iii)-imido species derived from aryl and alkyl azides capable of carrying out C–H bond activation. However, this system was not reported to be catalytic.^36^

In this manuscript we report the development of a cobalt catalyst for C–H amination that is tolerant of medicinally relevant heterocycles, such as pyridine and indole, and is capable of delivering 5-, 6-, and 7-membered rings. This combination of robust functional group tolerance and versatility with respect to ring size obtained, with low loadings of an earth-abundant transition metal complex, represents a significant advance in C–H amination catalysis.

**Fig. 1:**
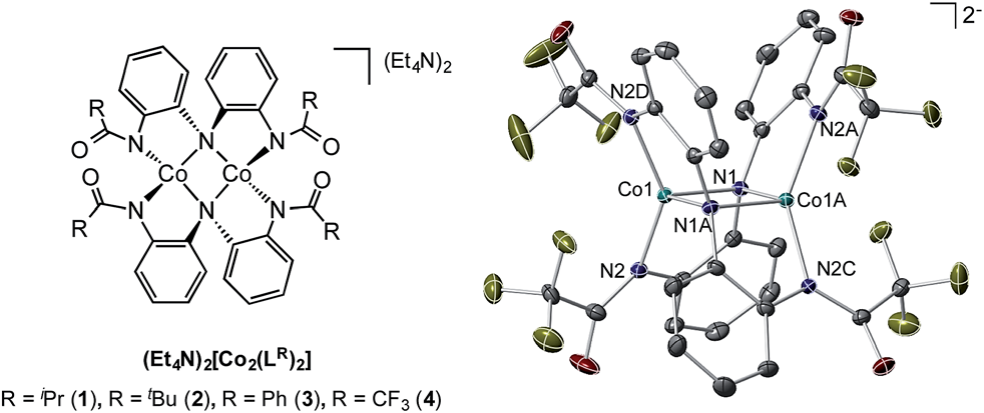
Left: family of dinuclear Co(ii) complexes investigated in this study (R = ^i^Pr, ^*t*^Bu, Ph, CF_3_); right: molecular structure of **4** determined by single crystal X-ray diffraction.

We have previously disclosed that Co(ii) complexes supported by a coordinatively versatile redox-active ligand derived from the NH(*o*-PhNHC(O)^i^Pr)_2_ scaffold are capable of promoting catalytic dioxygen activation to carry out O-atom transfer processes at impressive rates.^37^ The multi-electron processes required for this oxygen activation chemistry parallel those implicit in the azide activation/C–H amination sequence, suggesting that these novel dinuclear Co(ii) complexes should be investigated as catalysts for metallonitrene chemistry (Fig. 1).

## Results and discussion

*ortho*-Homobenzyl substituted aryl azide **5** was selected as a test substrate to explore the possibility of the cobalt(ii)-catalyzed intramolecular amination of aryl azides and establish effective reaction conditions (Table 1). No reaction was observed at room temperature with complex **1** and azide **5** over the course of 24 hours (entry 1). However, performing the reaction at elevated temperature (110 °C) produced indoline **6** as the only product in 43% yield (entry 2). To take advantage of the modularity of the ligand, dinuclear Co(ii) complexes with varying acyl substituents (R = CF_3_, Ph, ^*t*^Bu) were evaluated for catalytic activity for intramolecular C–H amination using aryl azide **5**. ^*t*^Butyl- and phenyl-substituted Co(ii) complexes **2** and **3** furnished lower indoline yields of 20% and 10%, respectively (entries 3 and 4). Substitution of an electron-withdrawing CF_3_ group on the ligand in complex **4** resulted in trace amounts of **6** (entry 5). Carrying out the reaction with lower catalyst loading (1 mol%) of the best-performing catalyst **1** resulted in a yield of 11% **6** over 24 hours (entry 6). Extending the reaction time to 48 hours resulted in an optimal yield of 83% **6** (entry 7). Control experiments show that under these reaction conditions azide **5** remains intact in the absence of a Co(ii) catalyst (entry 8). Furthermore, performing this reaction in the presence of a simple Co(ii) salt (CoBr_2_) resulted in no product formation (entry 9), suggesting that the ligand framework is essential to promote this transformation using Co(ii). It is important to note that this system is sensitive to water, and molecular sieves are required to achieve optimal indoline yields. In the absence of molecular sieves, a cyclized ligand byproduct (see ESI†) was observed, along with diminished indoline yields.

**Table 1:** Optimization of reaction conditions for C–H amination

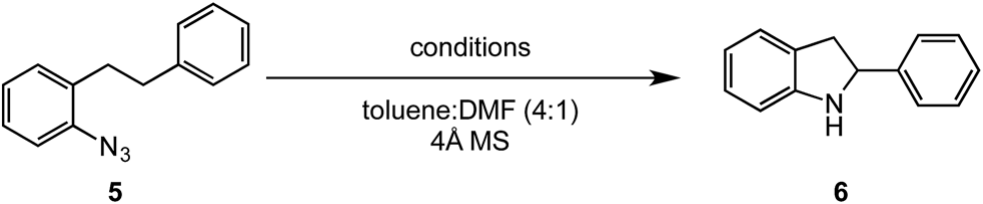					
Entry	Catalyst	Mol%	Time (h)	*T* (°C)	Yield^*a*^ (%)
1	**1**	10	24	25	0
2	**1**	10	24	110	43
3	**2**	10	24	110	20
4	**3**	10	24	110	10
5	**4**	10	24	110	*trace*
6	**1**	1	24	110	11
7	**1**	1	48	110	83
8	—	—	48	110	0
9	CoBr_2_	1	48	110	0

^*a*^Determined by ^1^H NMR using 1,3,5-trimethoxybenzene as an internal standard.

Under optimized conditions, azide **5** was converted to indoline in 80% isolated yield, notably without formation of the corresponding aniline or over-oxidation to the 2-phenylindole (Table 2, entry 1). The reaction was found to be robust with respect to the electronic nature of the homobenzylic aryl ring (83% and 81%, entries 2 and 3, respectively). Modulation of the aryl azide substitution resulted in a slight decrease in yield to 72% for CF_3_-substituted azide (entry 4). Notably, OMe substitution of the aryl azide resulted in 60% yield of 5-methoxy-2-phenylindole (entry 5). A 2 : 1 mixture of indoline to indole was observed by crude ^1^H NMR of the reaction mixture, but the indoline was oxidized upon exposure to silica gel chromatography during purification. The previously reported [(cod)Ir(OMe)]_2_ did not catalyze indoline formation with this particular substrate.^26^

**Table 2:** Scope of Co(ii)-catalyzed C–H amination with aryl azides

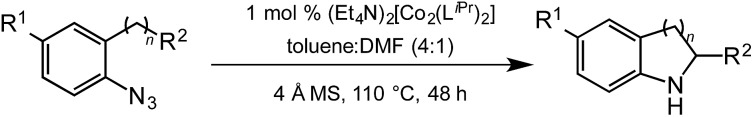			
Entry	Substrate	Product	Yield^*a*^
1	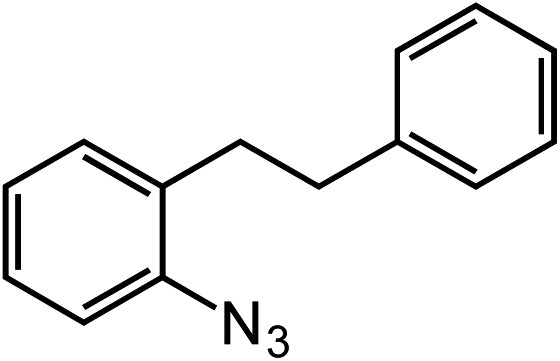	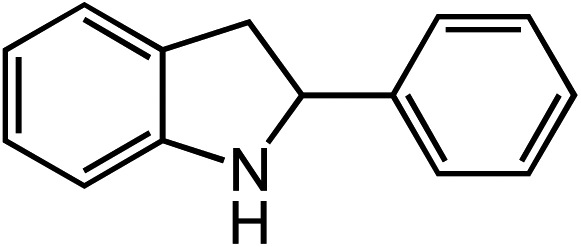	80
2	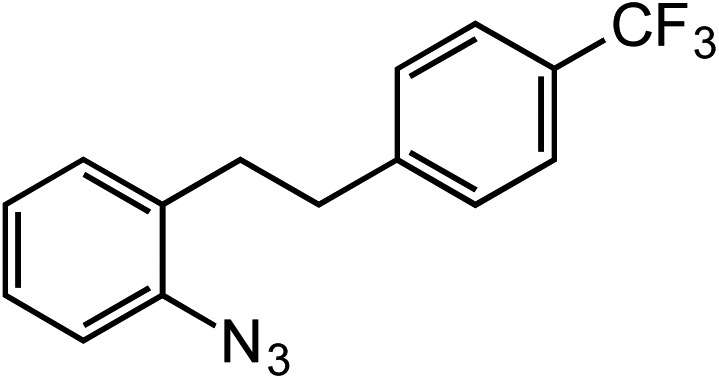	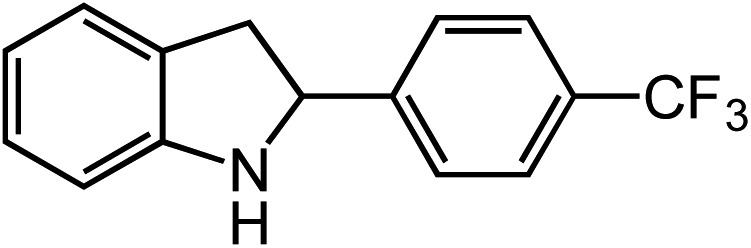	83
3	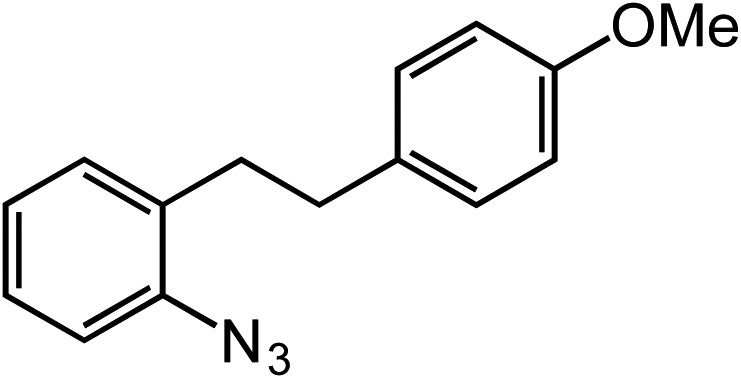	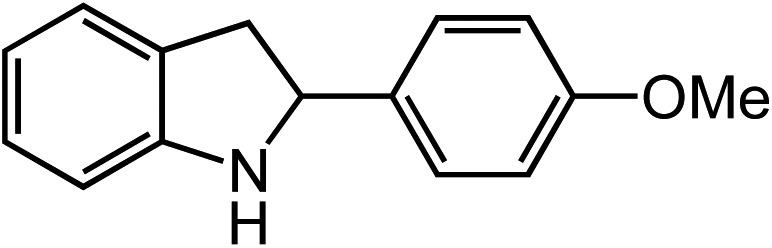	81
4	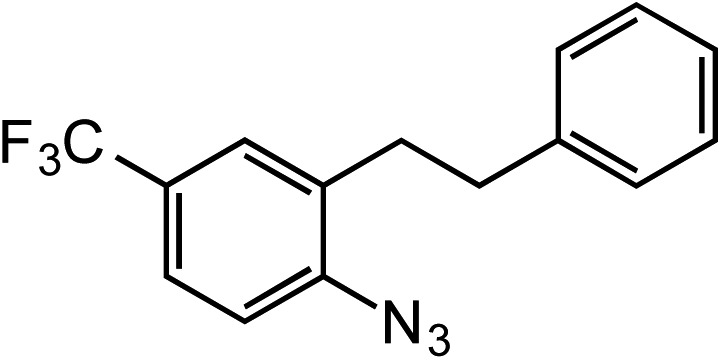	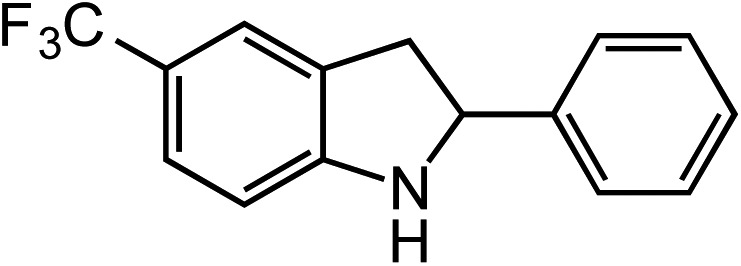	72
5	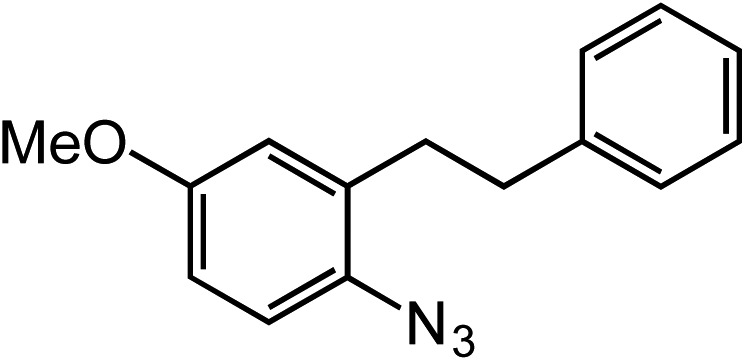	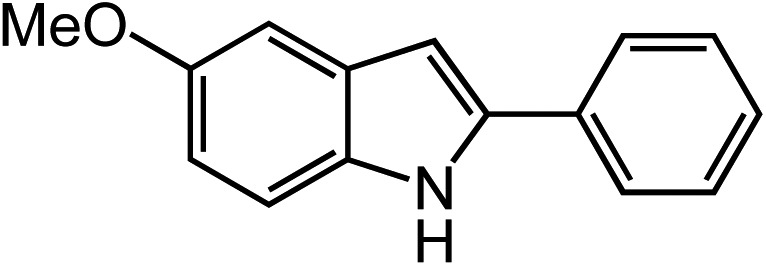	60^*b*^
6	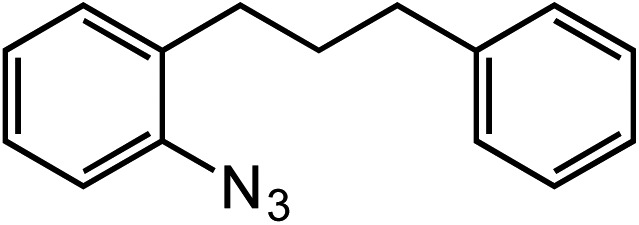	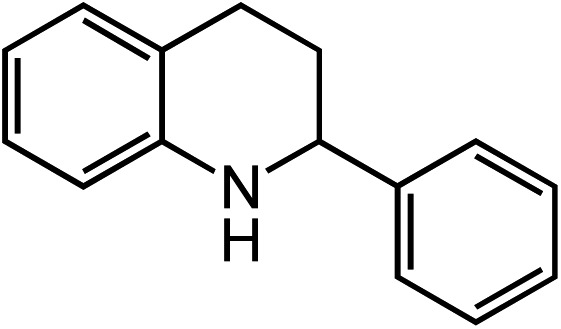	70
7	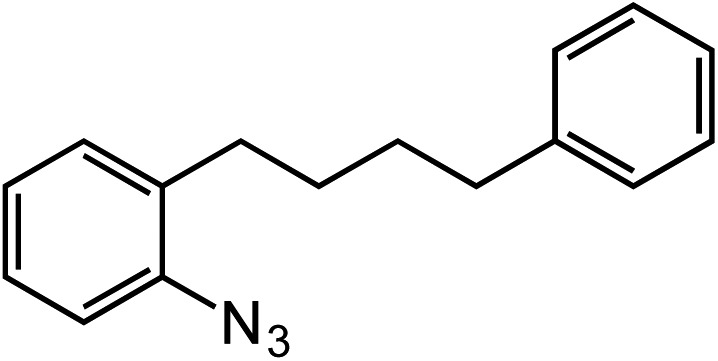	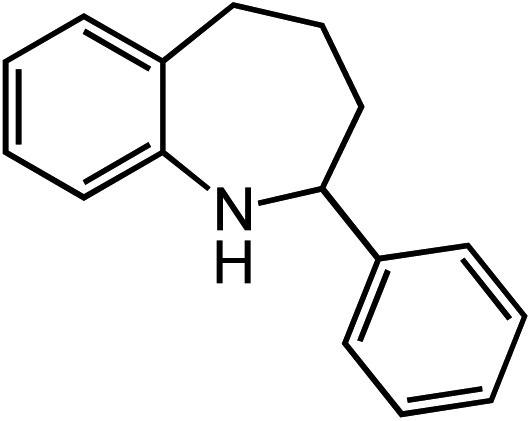	30^*c*^
8	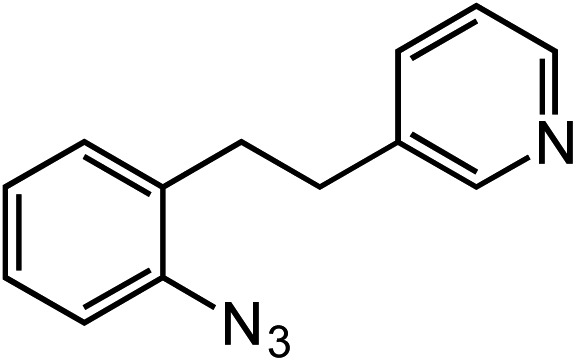	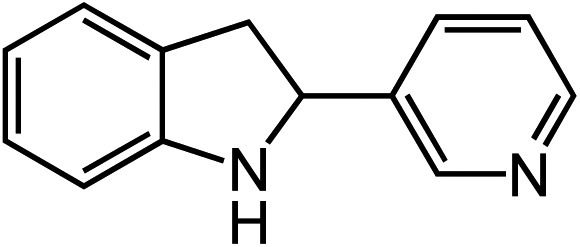	80
9	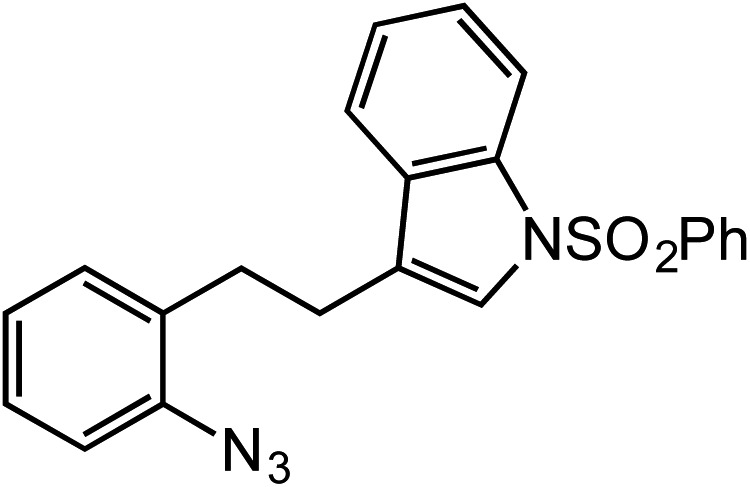	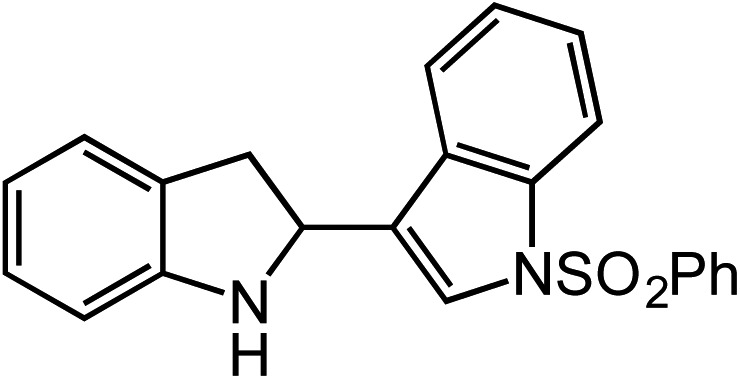	85
10	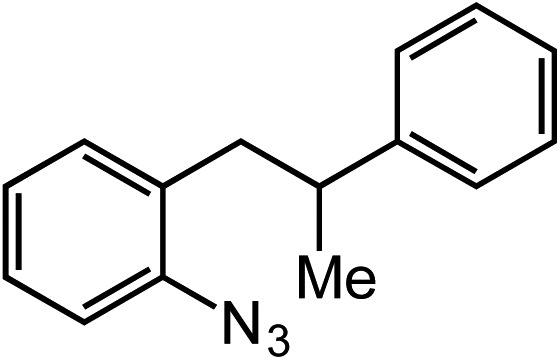	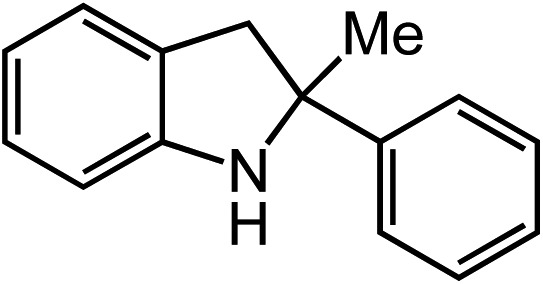	0^*d*^
11	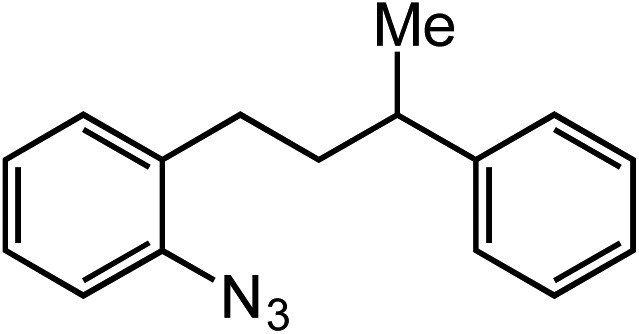	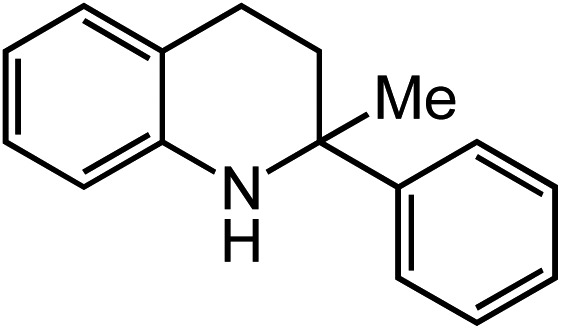	85

^*a*^Yield of isolated product.

^*b*^Yield of isolated indole.

^*c*^5 mol% catalyst loading.

^*d*^90% starting azide recovered from reaction mixture.

Extending the alkyl linker resulted in selective formation of the tetrahydroquinoline under these conditions, demonstrating six-membered ring formation is possible *via* this catalytic C–H amination reaction (Table 2, entry 6). Additionally, by extending the alkyl chain further, the seven-membered tetrahydrobenzoazepine was formed, albeit in diminished yield (30%, entry 7).

**Scheme 1:**
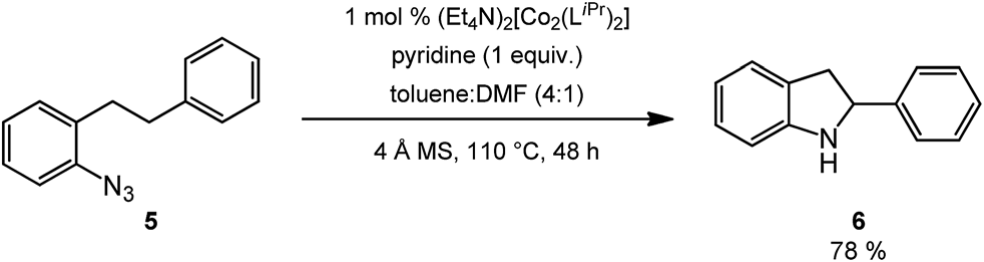
C–H amination in the presence of pyridine.

Novel heteroaromatic-containing substrates were also investigated with this catalyst system (Table 2, entries 8 and 9). Prior to investigating insertion at the benzylic position adjacent to pyridine, 1 equivalent of pyridine was added to the standard reaction of 1-azido-2-phenethylbenzene (**5**), and it was observed that the reaction proceeded normally (Scheme 1). This is notable due to the expectation that the high binding affinity of pyridine to transition metals might inhibit reactivity, as has been observed for other azide amination systems in the presence of exogenous coordinating ligands.^34^ Based on these results, the *ortho*-homopyridinyl-substituted aryl azide was synthesized and found to undergo amination in identical yield to the comparable benzylic insertion (entry 8). This tolerance of pyridine functionality, a privileged heterocycle for drug discovery, demonstrates the usefulness of this catalyst system for targets of pharmaceutical interest.^38^

In expanding to other biologically-relevant heteroaromatics, we investigated amination at the benzylic position adjacent to a protected indole and observed excellent conversion to product (85%, Table 2, entry 9). This also demonstrates the reaction is tolerant of an oxidation-sensitive substrate. However, in testing insertion into a benzylic 3° C–H center (Table 2, entry 10), we found amination was suppressed, resulting in 90% recovery of unreacted azide. Notably, when the alkyl chain was extended by one carbon, 3° C–H insertion was achieved to form the tetrahydroquinoline in 85% yield (Table 2, entry 11).

**Scheme 2:**
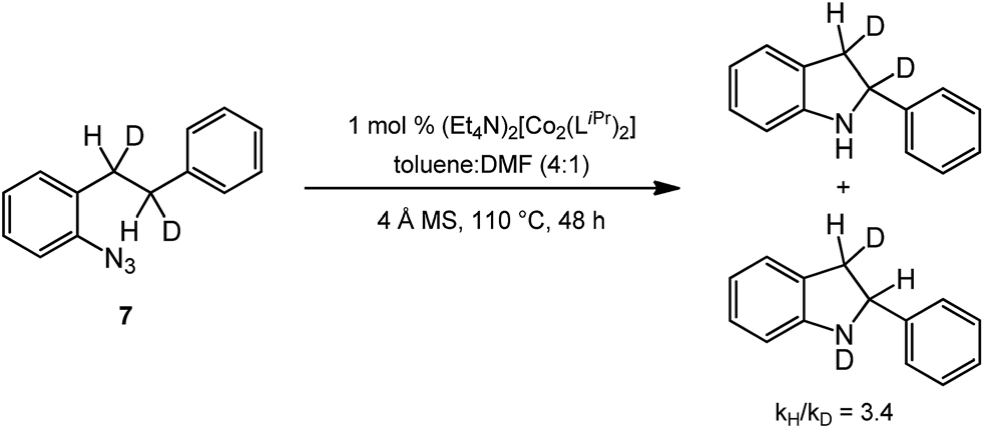
Kinetic isotope effect of intramolecular amination.

In order to probe the reaction pathway, the deuterated *ortho*-homobenzyl-aryl azide **7** was synthesized^27^ to measure the kinetic isotope effect (KIE) of this reaction. The deuterated substrate was subjected to the standard reaction conditions, and analysis of the crude reaction mixture by ^1^H NMR provided a KIE of 3.4 (Scheme 2). This kinetic isotope effect is lower than those observed for benzylic insertion by Driver and coworkers for the aryl azide system with [(cod)Ir(OMe)]_2_ (5.06)^26^ and Rh_2_(esp)_2_ (6.0)^27^ and by Betley and coworkers for their Fe-catalyzed alkyl azide amination (5.3).^34^ Zhang and coworkers observed a similarly greater value (6.2) for allylic insertion of a sulfonyl azide.^17^ While the value measured for the Co system is lower than these examples, it is higher than the KIE of 1.9 observed for dirhodium(ii) catalyzed intramolecular benzylic amination with sulfamate esters.^39^ These data suggest a hydrogen-atom abstraction/radical recombination mechanism maybe operative in this system. Further studies to propose a mechanism for these reactions are currently underway in our laboratories.

## Conclusions

In conclusion, we have shown a Co(ii) complex with a modular, redox-active ligand is capable of selectively catalyzing benzylic C–H amination with aryl azides utilizing a variety of substrates in good yields. This robust catalyst system holds potential for application in pharmaceutical and total synthesis, as well as extension to other substrate classes.

## Supplementary Material

Supplementary information

Crystal structure data
