# Supplementary material for: Cobalt catalyzed sp3 C–H amination utilizing aryl azides
Source: Chem Sci. 2015 Jul 30;6(11):6672–5. doi: 10.1039/c5sc01162k (PMC5802275; doi:10.1039/c5sc01162k)
Supplement: Supplementary file 1 [file SC-006-C5SC01162K-s001.pdf]

Electronic Supplementary Material (ESI) for Chemical Science.
This journal is © The Royal Society of Chemistry 2015

Cobalt catalyzed sp^3 C–H amination utilizing aryl azides

Omar Villanueva, Nina Mace Weldy, Simon B. Blakey and Cora E. MacBeth

Department of Chemistry, Emory University, Atlanta, GA, USA 30322

Supporting Information

Table of Contents

I. General Information 3
II. Procedures and Characterization Data
a) Syntheses of Ligands 4
b) Preparation of Cobalt(II) Complexes 5
c) Azides 6
d) Optimized Amination Reactions 10
e) Control Experiments 14
f) Cyclized Ligand Byproduct 14
III. Kinetic Isotope Effect Studies 16
IV. ^{1}H and ^{13}C NMR Spectra 19
V. X-Ray Crystallographic Data for Cobalt Complexes 36
VI. References 37

I. General Information.

^{1}H and ^{13}C NMR spectra were recorded at room temperature on a Varian Inova 600 spectrometer (600 MHz ^{1}H, 150 MHz ^{13}C) and a Varian Inova 400 spectrometer (400 MHz ^{1}H, 100 MHz ^{13}C) in $CDCl_3$ (neutralized and dried with anhydrous K_2CO_3) with internal $CHCl_3$ as the reference (7.27 ppm for ^{1}H and 77.23 ppm for ^{13}C). Chemical shifts (δ values) were reported in parts per million (ppm) and coupling constants (*J* values) in Hz. Multiplicity is indicated using the following abbreviations: s = singlet, d = doublet, t = triplet, q = quartet, qn = quintet, sext = sextet, m = multiplet, b = broad signal.). Solution-state infrared (IR) spectra were recorded using Thermo Electron Corporation Nicolet 380 FT-IR spectrometer. Solid-state Infrared spectra were recorded as KBr pellets on a Varian Scimitar 800 Series FT-IR spectrophotometer. UV-Visible absorption spectra were recorded on a Cary50 spectrophotometer using 1.0 cm quartz cuvettes. High-resolution mass spectra were obtained using a Thermo Electron Corporation Finigan LTQFTMS (Mass Spectrometry Facility, Emory University). X-ray diffraction studies were carried out in the X-ray Crystallography Laboratory at Emory University on a Bruker Smart 1000 CCD diffractometer. We acknowledge the use of shared instrumentation provided by grants from the NIH and the NSF. Elemental analyses were preformed by either Atlanta Microlab, Inc. Norcross, GA or Midwest Microlab, LLC, Indianapolis, IN. Analytical thin layer chromatography (TLC) was performed on precoated glass backed EMD 0.25 mm silica gel 60 plates. Visualization was accomplished with UV light or ethanolic anisaldehyde, followed by heating. Flash column chromatography was carried out using Silicycle® silica gel 60 (40-63 μm).

All reactions were conducted with anhydrous solvents in oven-dried and nitrogen-charged glassware or conducted in an MBraun Labmaster 130 drybox under a nitrogen atmosphere. Anhydrous solvents were purchased from Sigma-Aldrich and further purified by sparging with Ar gas followed by passage through activated alumina columns. Anhydrous solvents were stored inside an inert atmosphere dry box over freshly activated 4Å molecular sieves. Solvents for workup, extraction and column chromatography were used as received from commercial suppliers. All reagents used were purchased from commercial vendors and used as received unless otherwise noted.

II. Procedures and Characterization Data

a) Ligands

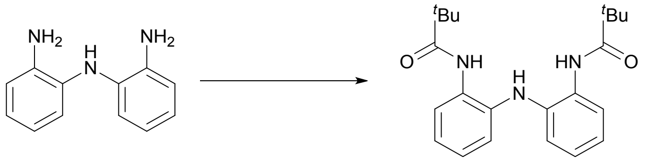

2,2'-Bis(*tert*-butylacetamido)diphenylamine (HN(*o*-PhNHC(O)^{t}Bu)$_2$) [H$_3$L^{tBu}]. A suspension of HN(*o*-PhNH$_2$)$_2$[1] (3.50 g, 17.6 mmol) in dichloromethane (DCM, 50 mL) was lowered to 0 °C under an atmosphere of N_2. Triethylamine (4.90 mL, 35.2 mmol) was then added, followed by the drop-wise addition pivaloyl chloride (4.30 mL, 35.2 mmol) via a dropper funnel. The mixture was stirred at 0 °C for 1 h. The reaction mixture was slowly warmed to room temperature and stirred for an additional 24 h. The resulting pale brown-colored solution was extracted with saturated aqueous $NaHCO_3$ (3 x 30 mL) and brine (2 x 20 mL), and the organic layer was dried over magnesium sulfate, filtered, and concentrated *in vacuo*. The crude solid was recrystallized by slow diffusion of diethyl ether into a concentrated DCM solution of the product (5.20 g, 80 %). **IR** (KBr, cm^{-1}) ν(NH)$_{Amine}$ 3343, ν(NH)$_{Amide}$ 3428, ν(CO) 1685; **^{1}H NMR** ($CDCl_3$, 400 MHz) δ 7.66 (dd, *J* = 7.6, 1.8 Hz, 2H), 7.64 (bs, 2H), 7.07-6.98 (m, 4H), 6.87 (dd, *J* = 7.8, 1.6 Hz, 2H), 6.08 (bs, 1H), 1.19 (s, 18H); **^{13}C NMR** ($CDCl_3$, 100 MHz) δ 177.3, 135.7, 129.2, 126.0, 123.5, 123.0, 121.0, 39.6, 27.5; **HRMS** (+ESI) calculated for $C_{22}H_{30}N_3O_2$ 368.23380, found 368.23305 $[M+H]^+$.

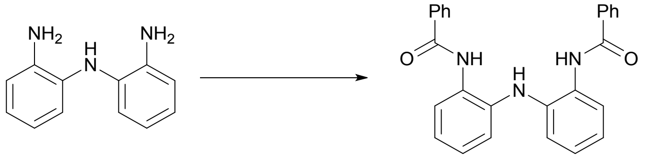

2,2'-Bis(diphenylacetamido)diphenylamine (HN(*o*-PhNHC(O)Ph)$_2$) [H$_3$L^{Ph}]. A suspension of HN(*o*-PhNH$_2$)$_2$[1] (6.31 g, 31.7 mmol) in dichloromethane (DCM, 50 mL) was lowered to 0 °C under an atmosphere of N_2. Triethylamine (9.75 mL, 63.4 mmol) was then added, followed by benzoyl chloride (8.10 mL, 63.4 mmol). The mixture was stirred at 0 °C for 1 h. The reaction mixture was slowly warmed to room temperature and stirred for an additional 20 h. The resulting pale brown solution was extracted with saturated aqueous $NaHCO_3$ (3 x 30 mL), and the organic layer was dried over magnesium sulfate, filtered, and concentrated *in vacuo*. The crude solid was recrystallized by layering hexanes over a concentrated DCM solution of the product (10.98 g, 85 %). **IR** (KBr, cm^{-1}) ν(NH) 3386, 3292, ν(CO) 1658; **^{1}H NMR** ($CDCl_3$, 400 MHz) δ 8.35 (s, 2H), 7.73 - 7.80 (m, 6H), 7.49 (t, 2H, *J* = 4.4 Hz), 7.38 (m, 4H), 7.13 -7.01 (m, 6H), 6.12 (s, 1H); **^{13}C NMR** ($CDCl_3$, 100 MHz) δ 166.4, 136.2, 134.2, 132.1, 129.1, 128.8, 127.6, 126.7, 124.5, 123.1, 121.0; **HRMS** (+ESI) calculated for $C_{26}H_{21}N_3O_2$ 407.1634, found 407.1626 $[M]^+$.

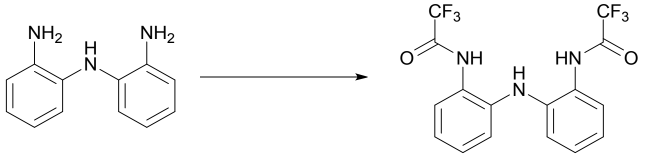

2,2'-Bis(trifluoroacetamido)diphenylamine (HN(*o*-PhNHC(O)CF$_3$)$_2$) [H$_3$L^{CF3}]. A suspension of HN(*o*-PhNH$_2$)$_2$[1] (2.55 g, 12.8 mmol) in dichloromethane (DCM, 50 mL) was lowered to 0 °C under

an atmosphere of N_2. Triethylamine (3.60 mL, 25.8 mmol) was then added, followed by trifluoroacetic anhydride (3.60 mL, 14.5 mmol). The mixture was stirred at 0 °C for 1 h. The reaction mixture was slowly warmed to room temperature and stirred for an additional 20 h. The resulting pale brown solution was extracted with saturated aqueous $NaHCO_3$ (3 x 30 mL), and the organic layer was dried over magnesium sulfate, filtered, and concentrated in vacuo. The crude solid was recrystallized by layering hexanes over a concentrated DCM solution of the product (1.78 g, 80 %). **IR** (KBr, cm^{-1}) ν(NH) 3298, ν(CO) 1701; **^{1}H NMR** ($CDCl_3$, 400 MHz) δ 8.33 (bs, 2H,), 7.67 (dd, 2H, *J* = 8.0, 1.6 Hz), 7.23 (td, 2H, *J* = 7.6, 1.6 Hz) 7.14 (td, 2H, *J* = 7.6, 1.2 Hz), 6.93 (dd, 2H, *J* = 8.0, 1.2 Hz), 5.49 (bs, 1H); **^{13}C NMR** ($CDCl_3$, 100 MHz) δ 131.9, 129.5, 127.7, 127.2, 125.3, 123.9, 1122.4, 110.7; **HRMS** (+ESI) calculated for $C_{16}H_{11}F_6N_3O_2$ 392.083, found 392.083 $[M+H]^+$.

b) Co(II) Complexes

The cobalt complexes are all prepared by similar procedures. The cobalt complexes can be prepared as either dipotassium or bistetraethylammonium salts. The choice of counter cation does not impact the amination reactions.

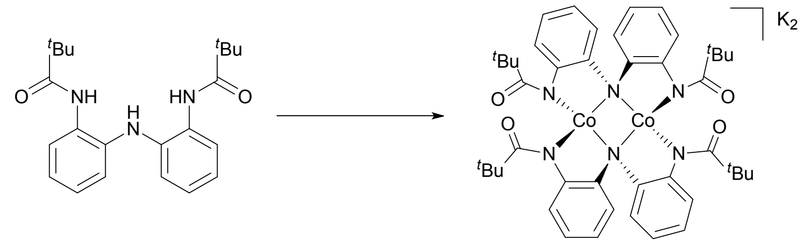

2

$K_2[Co_2(L^{tBu})_2]$ (2). To a solution of [HN(*o*-PhNHC(O)^{t}Bu)$_2$] (120 mg, 0.327 mmol) in dry dimethylformamide (DMF, 10 mL) was added potassium hydride (39.3 mg, 0.971 mmol) inside a nitrogen-filled atmosphere dry box. When gas evolution ceased, $CoBr_2$ (71 mg, 0.327 mmol) was added as a solid and the mixture stirred. After stirring for 3 h, DMF was removed under high vacuum and the resulting solid was dissolved in CH_3CN (15 mL), filtered through a medium porosity frit, and the filtrate was concentrated to dryness. Deep green X-ray diffraction quality crystals were obtained by slow diffusion of diethyl ether into a concentrated CH_3CN solution of $K_2[Co_2(L^{tBu})_2]$ (65 %, 179 mg). **IR** (KBr, cm^{-1}) ν(CO) 1664; **μ_{eff}** = 4.67(6) μB (Evans Method, CD_2Cl_2, 298K); **λ_{max}**, nm (ε, $M^{-1}cm^{-1}$) (CH_3CN): 587 (764), 820 (245) sh. **^{1}H NMR** ($CDCl_3$, 400 MHz) δ -65.65 (s), -48.87 (s), 14.20 (s), 18.214 (s), 28.21 (s), 59.39 (s); **HRMS** (+ESI) calculated for $[Co_2(L^{tBu})_2]^{1-}$ 846.271, found 846.273; Anal. Calcd (found) for $K_2[Co_2(L^{tBu})_2]$: C, 57.13 (56.63); H, 5.67 (5.49); N, 9.09 (9.44).

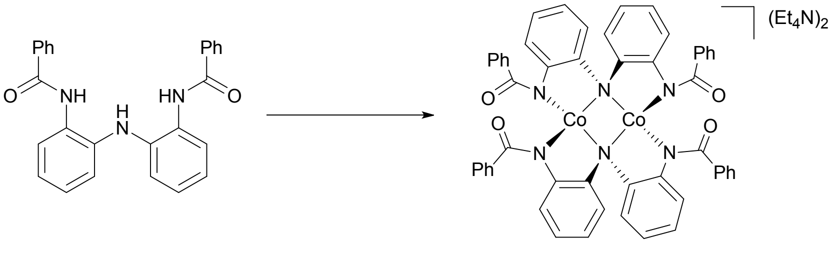

3

$(Et_4N)_2[Co_2(L^{Ph})_2]$ (3). To a solution of $[HN(o\text{-}PhNHC(O)Ph)_2]$ (150 mg, 0.368 mmol) in dry dimethylformamide (DMF, 10 mL) was added potassium hydride (44.3 mg, 1.10 mmol). When gas evolution ceased, $CoBr_2$ (81 mg, 0.368 mmol) was added as a solid and the mixture stirred for 1 h. Tetraethylammonium bromide (77.4 mg, 0.368 mmol) was then added to the deep green solution. After stirring for 3 h, DMF was removed under high vacuum and the resulting solid was dissolved in CH_3CN (15 mL), filtered through a medium porosity frit, and the filtrate was concentrated to dryness. Deep green X-ray diffraction quality crystals were obtained by slow diffusion of diethyl ether into a concentrated CH_3CN solution of $(Et_4N)_2[Co_2(L^{Ph})_2]$ (75 %, 164 mg). **IR** (KBr, cm^{-1}) ν(CO) 1655; μ_{eff} = 4.48(4) μB (Evans Method, CD_2Cl_2, 298K); λ_{max}, nm (ε, $M^{-1}cm^{-1}$) (CH_3CN): 597 (764), 936 (276); **^{1}H NMR** ($CDCl_3$, 400 MHz) δ -106.72 (s), -9.521 (s), -3.736 (s), 6.87 (s), 25.93 (s), 28.78 (s), 61.89 (s); **HRMS** (+ESI) calculated for $[Co_2(L^{Ph})_2]^{2-}$ 463.073, found 463.073; Anal. Calcd (found) for $(Et_4N)_2[Co_2(L^{Ph})_2]$•$CH_3CN$: C, 68.45 (68.62); H, 6.48 (6.18); N, 10.26 (10.36).

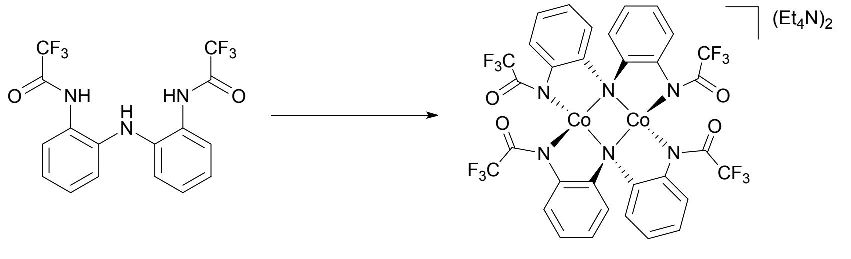

4

$(Et_4N)_2[Co_2(L^{CF3})_2]$ (4). To a solution of $HN(o\text{-}PhNHC(O)CF_3)_2$ (140 mg, 0.358 mmol) in anhydrous dimethylformamide (DMF, 10 mL) was added potassium hydride (43 mg, 1.08 mmol). When gas evolution ceased, $CoBr_2$ (78 mg, 0.358 mmol) was added as a solid, and the mixture was stirred for 1 h. Tetraethylammonium bromide (75 mg, 0.358 mmol) was then added to the deep magenta-teal solution. After stirring for 3 h, DMF was removed under high vacuum and the resulting solid was dissolved in CH_3CN (15 mL), filtered through a medium porosity frit, and the filtrate was concentrated to dryness. Deep teal X-ray diffraction quality crystals were obtained by slow diffusion of diethyl ether into a concentrated CH_3CN solution of $(Et_4N)_2[Co_2(L^{CF3})_2]$ (65%, 134 mg). **IR** (KBr, cm^{-1}) ν(CO) 1637; μ_{eff} = 4.82(7) μB (Evans Method, CD_3CN, 298K); λ_{max}, nm (ε, $M^{-1}cm^{-1}$) (CH_3CN): 597 (360), 876 (31); **^{1}H NMR** ($CDCl_3$, 400 MHz) δ) -1.372 (s), 38.753 (s), 44.278 (s), 54.962(s); **^{19}F NMR** (CD_3CN, 400 MHz) δ -57.139 (s); **HRMS** (-ESI) calculated for $(Et_4N)[Co_2(L^{CF3})_2]^-$ 1024.1310, found1024.16936, calculated for$[Co_2(L^{CF3})_2]^{2-}$ 446.986, found 446.99085; Anal. Calcd (found) for $(Et_4N)_2[Co_2(L^{CF3})_2)]$: C, 49.92 (48.88); H, 4.82 (4.72); N, 9.70 (9.30).

c) Azides

General Procedure A. Procedure from the literature[2] was modified as follows: alkyne (1.2 equiv.) was added to a solution of 2-bromoaniline (1.0 equiv.), $PdCl_2(PPh_3)_3$ (5 mol %), and CuI (2 mol %) in THF at room temperature. N_2 was bubbled through the stirring mixture for 30 mins, after which a solution of ethanolamine in H_2O (3.0 M, 12 equiv.) was added slowly. The mixture was heated to reflux until thin layer chromatography indicated complete consumption of starting material. The reaction was cooled to room temperature and extracted with DCM. The combined organic extracts were washed with brine, dried over Na_2SO_4, and concentrated *in vacuo*. Purification by flash chromatography on silica gel as indicated afforded the desired product.

General Procedure B. Aniline (1.0 equiv.) and Pd/C (10 wt. %, 0.1 equiv.) in THF (0.12 M) were stirred at room temperature under a balloon of H_2. After thin layer chromatography indicated complete consumption of the starting material, the mixture was filtered through Celite and washed with Et_2O to afford crude product.

$NaNO_2$ (1.2 equiv.) was added to a stirring solution of crude aniline (1.0 equiv.) in a 1:1 mixture of AcOH and H_2O (0.8 M) at 0 °C. The mixture was stirred for two hours, and NaN_3 (1.4 equiv.) was added. The reaction was warmed to room temperature. After 30 minutes, H_2O and Et_2O were added. Na_2CO_3 was added slowly to neutralize the solution until pH 7 was reached. The phases were separated, and the aqueous phase was extracted with Et_2O . The combined organic extracts were washed with brine, dried over Na_2SO_4, and concentrated *in vacuo*. Purification by flash chromatography on silica gel as indicated afforded the azide.

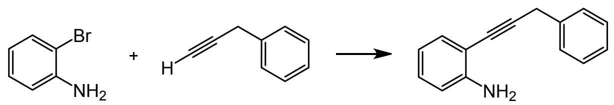

2-(3-phenylprop-1-yn-1-yl)aniline. Prepared by general procedure A using $PdCl_2(PPh_3)_3$ (211 mg, 0.3 mmol), CuI (22 mg, 0.12 mmol), 2-bromoaniline (1.04 g, 6.0 mmol), 3-phenyl-1-propyne (0.9 mL, 7.2 mmol), and ethanolamine (4.4 mL, 72 mmol). Purification by flash chromatography (95:5 hexanes/EtOAc) afforded the title compound as an orange oil (988 mg, 79 %); $\mathbf{R_f}$ 0.28 (9:1 hexanes/EtOAc); **IR** (thin film, cm^{-1}) 3474, 3378, 3028, 1612, 1492, 1307, 906, 729; **^{1}H NMR** ($CDCl_3$, 400 MHz) δ 7.43 (d, 2H, *J* = 8.2 Hz), 7.37-7.24 (m, 4H), 7.11 (t, 1H, *J* = 7.6 Hz) 6.71-6.67 (m, 2H), 4.14 (bs, 2H), 3.90 (s, 2H); **^{13}C NMR** ($CDCl_3$, 100 MHz) δ 147.8, 136.8, 132.2, 129.1, 128.6, 127.9, 126.7, 117.8, 114.2, 108.4, 92.9, 79.2, 26.0; **HRMS** (+ESI) calculated for $C_{15}H_{14}N$ 208.1121, found 208.1117 $[M+H]^+$.

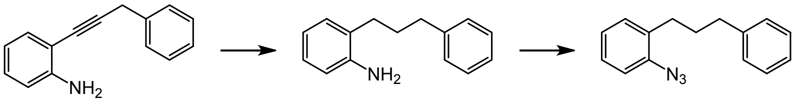

1-azido-2-(3-phenylpropyl)benzene. Prepared by general procedure B using 2-(3-phenylprop-1-yn-1-yl)aniline (947 mg, 4.6 mmol), Pd/C (450 mg, 10 wt. %), $NaNO_2$ (380 mg, 5.5 mmol), and NaN_3 (420 mg, 6.6 mmol). Purification by flash chromatography (hexanes → 97:3 hexanes/EtOAc) afforded the title compound as a light yellow oil (410 mg, 38 % over two steps); $\mathbf{R_f}$ 0.85 (95:5 hexanes/EtOAc); **IR** (thin film, cm^{-1}) 3025, 2990, 2858, 2116, 1581, 1488, 1450, 1282, 745; **^{1}H NMR** ($CDCl_3$, 400 MHz) δ 7.29-7.22 (m, 3H), 7.21-7.10 (m, 5H), 7.04 (t, 1H, *J* = 7.4 Hz), 2.66-2.58 (m, 4H), 1.89 (quin, 2H, *J* = 7.8 Hz); **^{13}C NMR** ($CDCl_3$, 100 MHz) δ 142.2, 138.0, 133.8, 130.4, 128.5, 128.3, 127.3, 125.8, 124.7, 118.1, 35.7, 31.9, 31.0; **HRMS** (+ESI) calculated for $C_{15}H_{18}N$ 212.1434, found 212.1431 $[M-2N+3H]^+$.

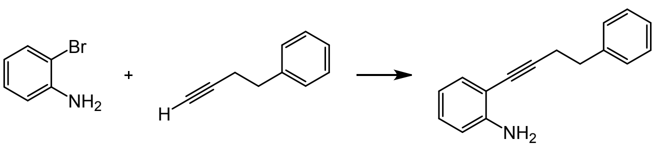

2-(4-phenylbut-1-yn-1-yl)aniline. Prepared by general procedure A using $PdCl_2(PPh_3)_3$ (416 mg, 0.59 mmol), CuI (45 mg, 0.24 mmol), 2-bromoaniline (2.04 g, 11.9 mmol), 4-phenyl-1-butyne (2.0 mL, 14.2 mmol), and ethanolamine (8.6 mL, 142 mmol). Purification by flash chromatography (95:5 hexanes/EtOAc) afforded the title compound as an orange oil (820 mg,

31 %); $\mathbf{R_f}$ 0.28 (9:1 hexanes/EtOAc); **IR** (thin film, cm^{-1}) 3471, 3375, 3026, 2925, 1612, 1491, 1454, 1307, 905, 744; ^{1}**H NMR** ($CDCl_3$, 400 MHz) δ 7.32-7.27 (m, 4H), 7.24-7.19 (m, 2H), 7.05 (td, 1H, *J* = 7.7, 1.3 Hz) 6.63 (t, 2H, *J* = 7.5 Hz), 3.95 (bs, 2H), 2.93 (t, 2H, *J* = 7.5 Hz), 2.78 (t, 2H, *J* = 7.5 Hz); ^{13}**C NMR** ($CDCl_3$, 100 MHz) δ 147.8, 140.6, 131.9, 128.9, 128.6, 128.4, 126.3, 117.7, 114.1, 108.5, 94.6, 77.9, 35.1, 21.7; **HRMS** (+ESI) calculated for $C_{16}H_{16}N$ 222.1277, found 222.1273 $[M+H]^+$.

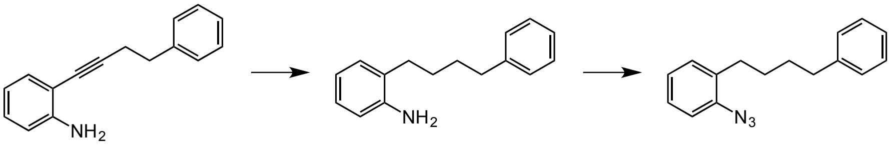

1-azido-2-(4-phenylbutyl)benzene. Prepared by general procedure B using 2-(4-phenylbut-1-yn-1-yl)aniline (820 mg, 3.7 mmol), Pd/C (370 mg, 10 wt. %), $NaNO_2$ (306 mg, 4.4 mmol), and NaN_3 (331 mg, 5.2 mmol). Purification by flash chromatography (hexanes → 97:3 hexanes/EtOAc) afforded the title compound as a light yellow oil (383 mg, 41 % over two steps); $\mathbf{R_f}$ 0.87 (95:5 hexanes/EtOAc); **IR** (thin film, cm^{-1}) 3024, 2929, 2857, 2113, 1581, 1488, 1451, 1283, 747; ^{1}**H NMR** ($CDCl_3$, 400 MHz) δ 7.29-7.10 (m, 8H), 7.03 (td, 1H, *J* = 7.0, 1.2 Hz), 2.65-2.56 (m, 4H), 1.69-1.55 (m, 4H); ^{13}**C NMR** ($CDCl_3$, 100 MHz) δ 142.5, 137.9, 134.0, 130.4, 128.4, 128.2, 127.2, 125.6, 124.6, 118.0, 35.8, 31.2, 31.0, 29.9; **HRMS** (+ESI) calculated for $C_{16}H_{20}N$ 226.1590, found 226.1586 $[M-2N+3H]^+$.

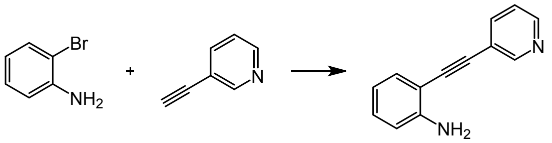

2-(pyridin-3-ylethynyl)aniline. Prepared by general procedure A using $PdCl_2(PPh_3)_3$ (550 mg, 0.78 mmol), CuI (73 mg, 0.38 mmol), 2-bromoaniline (1.9 mL, 16.8 mmol), 3-ethynylpyridine (2.1 g, 20.4 mmol), and ethanolamine (12.3 mL, 204 mmol). Purification by flash chromatography (9:1 → 8:2 hexanes/EtOAc) afforded the title compound as an orange oil (1.78 g, 54 %); $\mathbf{R_f}$ 0.20 (7:3 hexanes/EtOAc); **IR** (thin film, cm^{-1}) 3456, 3324, 3192, 3030, 2211, 1615, 1493, 1407, 1314, 748; ^{1}**H NMR** ($CDCl_3$, 400 MHz) δ 8.75 (dd, 1H, *J* = 2.3, 0.8 Hz), 8.53 (dd, 1H, *J* = 4.9, 1.8 Hz), 7.78 (dt, 1H, *J* = 7.8, 2.0 Hz), 7.37 (dd, 1H, *J* = 8.0, 1.4 Hz), 7.27 (ddd, 1H, *J* = 8.4, 4.3, 1.0 Hz), 7.16 (ddd, 1H, *J* = 8.1, 7.1, 1.6 Hz), 6.74-6.70 (m, 2H), 4.31 (bs, 2H); ^{13}**C NMR** ($CDCl_3$, 100 MHz) δ 152.03, 148.5, 147.9, 138.2, 132.2, 130.3, 123.0, 120.5, 118.0, 114.4, 107.1, 91.2, 89.3; **HRMS** (+ESI) calculated for $C_{13}H_{11}N_2$ 195.0917, found 195.0914 $[M+H]^+$.

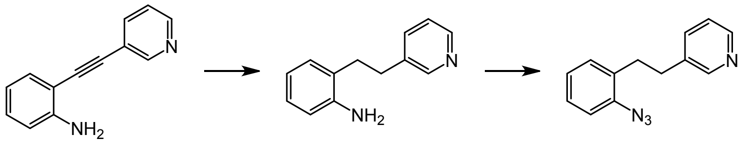

3-(2-azidophenethyl)pyridine. Prepared by general procedure B using 2-(pyridin-3-ylethynyl)aniline (1.5 g, 7.73 mmol), Pd/C (880 mg, 10 wt. %), $NaNO_2$ (640 mg, 9.28 mmol), and NaN_3 (700 mg, 10.94 mmol). Purification by flash chromatography (8:2 hexanes/EtOAc) afforded the title compound as a light yellow oil (1.32 g, 76 % over two steps); $\mathbf{R_f}$ 0.35 (7:3 hexanes/EtOAc); **IR** (thin film, cm^{-1}) 3027, 2928, 2118, 1709, 1579, 1489, 1284, 906, 712; ^{1}**H NMR** ($CDCl_3$, 400 MHz) δ 8.42 (tt, 2H, *J* = 5.1, 1.2 Hz), 7.47 (d, 1H, *J* = 7.9 Hz), 7.27-7.23

(m, 1H), 7.20 (dd, 1H, J = 7.8, 4.7 Hz), 7.13 (d, 1H, J = 7.9 Hz), 7.06-7.0 (m, 2H), 2.85 (s, 4H); **^{13}C NMR** ($CDCl_3$, 100 MHz) δ 149.7, 147.3, 137.9, 136.8, 136.1, 132.1, 130.5, 127.7, 124.7, 123.2, 118.1, 33.5, 32.9; **HRMS** (+ESI) calculated for $C_{13}H_{13}N_4$ 225.1135, found 225.1134 $[M+H]^+$.

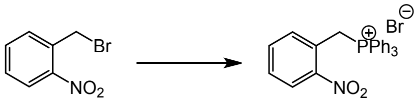

Phosphonium salt. Procedure from the literature[3] was modified as follows: to a solution of 2-nitrobenzylbromide (10.1 g, 46.9 mmol, 1 equiv.) in DMF (33 mL) was added triphenylphosphine (12.3, g, 46.9 mmol, 1 equiv.). The reaction mixture was stirred 26 h at room temperature, at which point it was diluted with toluene (1 M) and filtered. The filter was washed with DCM to dissolve the solid filtrate. The product solution was concentrated *in vacuo* and recrystallized out of DCM/Et_2O to yield pure phosphonium salt as a white solid (21.2 g, 94 %); **IR** (thin film, cm^{-1}) 3420, 3058, 2856, 2160, 1525, 1437, 1340, 1110, 722; **^{1}H NMR** ($CDCl_3$, 400 MHz) δ 8.12 (dt, 1H, 7.5, 3.1 Hz), 7.91 (d, 1H, J = 8.3 Hz), 7.77 (td, 3H, J = 7.5, 0.9 Hz) 7.68 (dd, 6H, J = 12.7, 8.3 Hz), 7.63-7.59 (m, 7H), 7.46 (tt, 1H, J = 7.5, 2.2 Hz) 6.13 (d, 1H, J = 14.9 Hz); **^{13}C NMR** ($CDCl_3$, 100 MHz) δ 135.2, 134.3, 134.2, 130.3, 130.2, 125.7, 117.6, 117.0, 28.7; **HRMS** (+ESI) calculated for $C_{25}H_{21}NO_2P$ 398.1310, found 398.1282 $[M-Br^-]^+$.

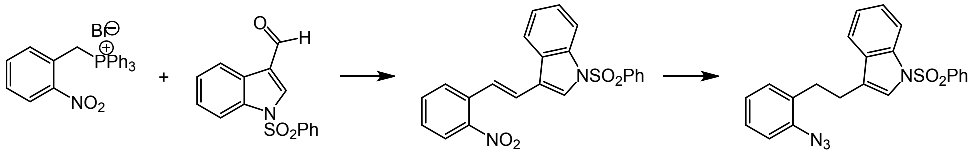

3-(2-azidophenethyl)-1-(phenylsulfonyl)-1H-indole. Procedure from the literature[3] was modified as follows: a solution of phosphonium salt (2.04 g, 4.3 mmol, 1.2 equiv.) in THF (0.2 M) was cooled to 0 °C, and KO^{t}Bu (630 mg, 5.6 mmol, 1.6 equiv.) was added. The solution was stirred for 30 min at 0 °C, and 1-(phenylsulfonyl)-3-indolecarboxaldehyde (980 mg, 3.5 mmol, 1.0 equiv.) was added. The mixture was warmed to room temperature overnight and quenched with H_2O and EtOAc after 22 h. The phases were separated, and the aqueous phase was extracted with EtOAc. The combined organic extracts were washed with brine, dried over $MgSO_4$, and concentrated *in vacuo*. The crude mixture was dissolved in EtOAc and passed through a plug of silica. The crude filtrate was concentrated *in vacuo* and carried on through general procedure B using Pd/C (300 mg, 10 wt. %), $NaNO_2$ (100 mg, 1.43 mmol), and NaN_3 (113 mg, 1.67 mmol). Purification by flash chromatography (8:2 → 7:3 hexanes/EtOAc) afforded the title compound as an orange solid (320 mg, 44 % over three steps); **R_f** 0.7 (7:3 hexanes/EtOAc); **IR** (thin film, cm^{-1}) 3065, 2928, 2119, 1446, 1365, 1280, 1173, 905, 725; **^{1}H NMR** ($CDCl_3$, 400 MHz) δ 7.97 (dd, 1H, J = 8.2, 0.8 Hz), 7.81 (d, 2H, J = 8.6 Hz); 7.52 (t, 2H, J = 7.4 Hz), 7.41 (t, 2H, J = 7.4 Hz), 7.32-7.21 (m, 4H), 7.13 (d, 1H, J = 8.2), 7.05-6.97 (m, 2H), 2.91 (s, 4H); **^{13}C NMR** ($CDCl_3$, 100 MHz) δ 138.3, 138.0, 135.3, 133.6, 132.7, 131.0, 130.5, 129.2, 127.6, 126.7, 124.7, 123.1, 122.8, 122.7, 119.5, 118.1, 113.7, 31.0, 25.6; **HRMS** (+ESI) calculated for $C_{22}H_{21}O_2N_2S$ 377.1318, found 377.1319 $[M-2N+3H]^+$.

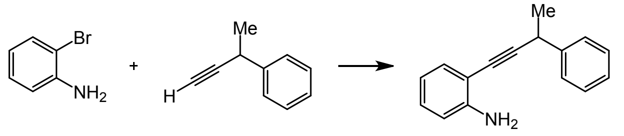

2-(3-phenylbut-1-yn-1-yl)aniline. Prepared by general procedure A using $PdCl_2(PPh_3)_3$ (211 mg, 0.3 mmol), CuI (23 mg, 0.12 mmol), 2-bromoaniline (1.04 g, 6.0 mmol), (1-methyl-2-propyn-1-yl)-benzene[4] (940 mg, 7.2 mmol), and ethanolamine (4.4 mL, 72 mmol). Purification by flash chromatography (95:5 hexanes/EtOAc) afforded the title compound as an orange oil (498 mg, 37 %); $\mathbf{R_f}$ 0.31 (95:5 hexanes/EtOAc); **IR** (thin film, cm^{-1}) 3472, 3378, 3027, 2974, 1611, 1491, 1453, 1305, 747; **^{1}H NMR** ($CDCl_3$, 400 MHz) δ 7.44 (d, 2H, *J* = 7.0 Hz), 7.33 (tt, 2H, *J* = 7.7, 1.76), 7.27-7.22 (m, 2H), 7.07 (td, 1H, *J* = 7.8, 1.5 Hz), 6.67-6.64 (m, 2H), 4.13 (bs, 2H), 4.03 (q, 1H, *J* = 7.0 Hz), 1.59 (d, 3H, *J* = 7.0 Hz); **^{13}C NMR** ($CDCl_3$, 100 MHz) δ 147.7, 143.4, 132.1, 129.1, 128.6, 126.9, 126.7, 117.8, 114.2, 108.5, 98.1, 78.9, 32.8, 24.8; **HRMS** (+ESI) calculated for $C_{16}H_{16}N$ 222.1277, found 222.1279 $[M+H]^+$.

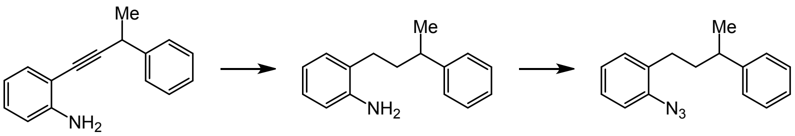

1-azido-2-(3-phenylpropyl)benzene. Prepared by general procedure B using 2-(3-phenylbut-1-yn-1-yl)aniline (438 mg, 2.0 mmol), Pd/C (200 mg, 10 wt. %), $NaNO_2$ (166 mg, 2.4 mmol), and NaN_3 (180 mg, 2.8 mmol). Purification by flash chromatography (hexanes → 97:3 hexanes/EtOAc) afforded the title compound as a light yellow oil (371 mg, 75 % over two steps); $\mathbf{R_f}$ 0.9 (95:5 hexanes/EtOAc); **IR** (thin film, cm^{-1}) 2958, 2116, 1489, 1450, 1283, 905, 727; **^{1}H NMR** ($CDCl_3$, 400 MHz) δ 7.32-7.28 (m, 2H), 7.22-7.18 (m, 4H), 7.08 (td, 2H, *J* = 8.0, 1.6 Hz), 7.01 (td, 1H, *J* = 7.4, 1.2 Hz), 2.71 (sxt, 1H, *J* = 7.1 Hz), 2.53-2.37 (m, 2H), 1.87-1.78 (m, 2H), 1.27 (d, 3H, *J* = 7.0 Hz); **^{13}C NMR** ($CDCl_3$, 100 MHz) δ 147.2, 137.9, 134.0, 130.3, 128.4, 127.2, 127.1, 126.0, 124.6, 118.0, 39.8, 38.6, 29.5, 22.6; **HRMS (+ESI)** calculated for $C_{15}H_{18}N$ 212.1434, found 212.1431 $[M-2N+3H]^+$.

d) Amination Reactions

General Procedure C. Inside a nitrogen-filled atmosphere dry box, a 20 mL scintillation vial was charged with a magnetic stir bar, aryl azide (1.0 equiv.), $\mathbf{(Et_4N)_2[Co_2(L^{iPr})_2]}$ (1 mol%)[1], and a 4:1 mixture of toluene and DMF, respectively. To the reaction mixture was added freshly activated 4 Å molecular sieves. The reaction vial was sealed with a Teflon cap and the reaction mixture was heated to 110 °C for 24 h inside the dry box on a DynaBloc heating block. After 24 h, the reaction vial was removed from the heating block and allowed to stir at room temperature for 30 minutes. After reaction mixture cooled to room temperature, the molecular sieves were removed by filtration, and the solvent was removed under vacuum. Purification by flash chromatography on silica gel (7:1 hexanes/EtOAc) as indicated afforded the desired product.

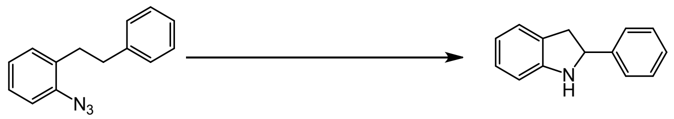

5 **6**

2-phenylindoline (6).[2] Prepared by general procedure C using azide **5**[2] (150 mg, 0.672 mmol) and $\mathbf{(Et_4N)_2[Co_2(L^{iPr})_2]}$[1] (7.0 mg, 0.007 mmol) in 5.0 mL of a 4:1 mixture of toluene and DMF, respectively. Purification by flash chromatography (7:1 hexanes/EtOAc) afforded the title compound (105 mg, 80 %); ^{1}H NMR (400 MHz, $CDCl_3$) δ 7.44 - 7.28 (m, 5H), 7.12 - 7.04 (m,

2H), 6.76 (td, J = 1.0, 7.4 Hz, 1H), 6.69 (d, J = 7.3 Hz, 1H), 4.97 (t, J = 9.0 Hz, 1H), 4.17 (s, 1H), 3.47 (dd, J = 9.2, 15.7 Hz, 1H), 3.00 (dd, J = 8.8, 15.7 Hz, 1H).

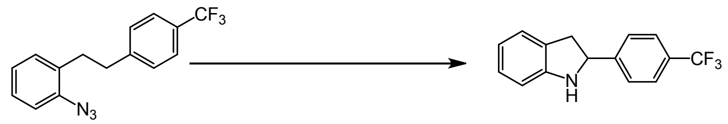

2-(4-(trifluoromethyl)phenyl)indoline.[2] Prepared by general procedure C using 1-azido-2-(4-(trifluoromethyl)phenethyl)benzene[2] (150 mg, 0.515 mmol) and **$(Et_4N)_2[Co_2(L^{iPr})_2]$** (5.0 mg, 0.005 mmol) in 5.0 mL of a 4:1 mixture of toluene and DMF, respectively. Purification by flash chromatography (7:1 hexanes/EtOAc) afforded the title compound (113 mg, 83 % yield). ^{1}H NMR (400 MHz, $CDCl_3$) δ 7.63 - 7.50 (m, 4H), 7.08 (m, 2H), 6.74 (tdd, J = 7.4, 3.6, 1.1 Hz, 1H), 6.69 (d, J = 7.6 Hz, 1H), 5.01 (t, J = 9.3 Hz, 1H), 4.16 (s, 1H), 3.47 (dd, J = 15.6, 9.1 Hz, 1H), 2.93 (dd, J = 15.6, 9.1 Hz, 1H).

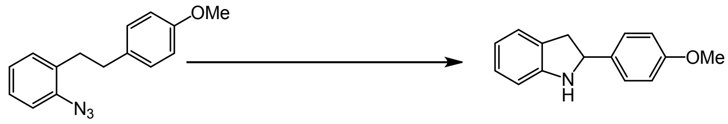

2-(4-methoxyphenyl)indoline.[2] Prepared by general procedure C using 1-azido-2-(4-methoxyphenethyl)benzene[2] (175 mg, 0.691 mmol) and **$(Et_4N)_2[Co_2(L^{iPr})_2]$[1]** (7.3 mg, 0.0069 mmol) in 5.0 mL of a 4:1 mixture of toluene and DMF, respectively. Purification by flash chromatography (7:1 hexanes/EtOAc) afforded the title compound (126 mg, 81 % yield). ^{1}H NMR (400 MHz, $CDCl_3$) δ 7.35 (d, J = 8.4 Hz, 2H), 7.11 - 7.06 (m, 2H), 6.88 (d, J = 8.0 Hz, 2H), 6.76 - 6.73 (m, 1H), 6.67 (d, J = 8 Hz, 1H) 4.91 (t, J = 9.0 Hz, 1H), 4.11 (s, 1H), 3.81 (s, 3H), 3.41 (dd, J = 16.8, 6.4 Hz, 1H), 2.97 (dd, J = 17.6, 6.8 Hz, 1H).

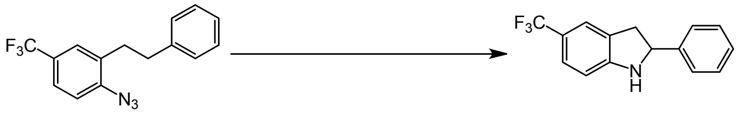

2-phenyl-5-(trifluoromethyl)indoline.[2] Prepared by general procedure C using 1-azido-2-phenethyl-4-(trifluoromethyl)benzene[2] (170 mg, 0.584 mmol) and **$(Et_4N)_2[Co_2(L^{iPr})_2]$**[1] (6.1 mg, 0.0061 mmol) in 5.0 mL of a 4:1 mixture of toluene and DMF, respectively. Purification by flash chromatography (7:1 hexanes/EtOAc) afforded the title compound (110 mg, 72 % yield). ^{1}H NMR (400 MHz, $CDCl_3$) δ 7.50 - 7.59 (m, 7H), 6.69 (d, J = 8.0 Hz, 1H), 5.04 (t, J = 9.0 Hz, 1H), 4.40 (s, 1H), 3.50 (dd, J = 15.6, 9.4 Hz, 1H), 3.00 (dd, J = 15.8, 8.4 Hz, 1H).

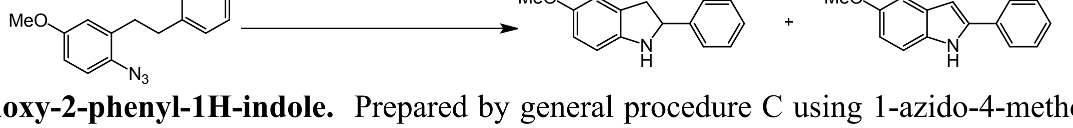

5-methoxy-2-phenyl-1H-indole. Prepared by general procedure C using 1-azido-4-methoxy-2-phenethylbenzene[2] (160 mg, 0.632 mmol) and **$(Et_4N)_2[Co_2(L^{iPr})_2]$**[1] (6.0 mg, 0.006 mmol) in 5.0 mL of a 4:1 mixture of toluene and DMF, respectively. Analysis of ^{1}H NMR before purification revealed indoline and indole were formed in a 2:1 ratio. Purification by flash chromatography (7:1 hexanes/EtOAc) resulted in recovery of 30 % 1-azido-4-methoxy-2-phenethylbenzene (48 mg), and exclusive formation of the oxidized indole[5] product (85 mg, 60 % yield). ^{1}H NMR (400 MHz, $CDCl_3$) δ 8.22 (bs, 1H), 7.63 (dd, J = 10 Hz, 2H) 7.47 - 7.37

(m, 2H), 7.08 (d, J = 2.5 Hz, 1H), 6.85 (td, J = 8.8, 2.4 Hz, 1H), 6.75 (dt, J = 2.3, 1.2 Hz, 1H), 3.86 (s, 3H).

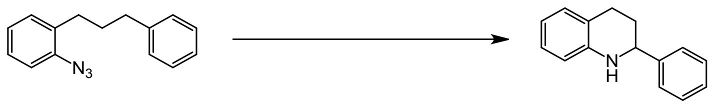

2-phenyl-1,2,3,4-tetrahydroquinoline. Prepared by general procedure C using 1-azido-2-(4-phenylbutyl)benzene (150 mg, 0.632 mmol) and **$(Et_4N)_2[Co_2(L^{iPr})_2]$**[1] (6.6 mg, 0.0063 mmol) in 5.0 mL of a 4:1 mixture of toluene and DMF, respectively. Purification by flash chromatography (7:1 hexanes/EtOAc) afforded the title compound (93 mg, 70 % yield).[6] ^{1}H NMR (400 MHz, $CDCl_3$) δ 7.26-7.39 (m, 5H), 6.97-6.99 (m, 2H), 6.64 (td, J = 7.6, 0.8 Hz, 1H), 6.51 (d, J = 7.6 Hz, 1H), 4.41 (dd, J = 9.2, 3.2 Hz, 1H), 2.88-2.90 (m, 1H), 2.74 (dt, J = 16.4, 4.8 Hz, 1H), 2.08-2.10 (m, 1H), 1.96-2.02 (m, 1H).

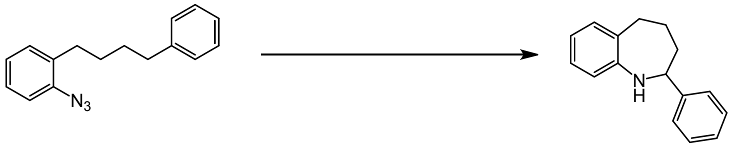

2-phenyl-2,3,4,5-tetrahydro-1H-benzo[b]azepine. General procedure C was modified as follows: a mixture of 1-azido-2-(4-phenylbutyl)benzene (75 mg, 0.299 mmol) and 5 mol % **$(Et_4N)_2[Co_2(L^{iPr})_2]$**[1] (16 mg, 0.0076 mmol) was stirred in a 5.0 mL of a 4:1 mixture of toluene and DMF, respectively. Purification by flash chromatography (9:1 hexanes/EtOAc) afforded the title compound as a light orange oil (20 mg, 30 % yield). **R_f** (9:1 hexanes/EtOAc) 0.3; **IR** (thin film, cm^{-1}) 3840, 3648, 2924, 2702, 2356, 2157, 1935, 903, 721; **^{1}H NMR** ($CDCl_3$, 400 MHz) δ 7.29-7.17 (m, 5H), 7.07-7.05 (m, 1H), 6.99 (t, J = 7.6 Hz, 1H), 6.67 (t, J = 7.3 Hz, 1H), 6.58 (d, J = 7.6 Hz, 1H), 3.89-3.84 (m, 2H), 3.14 (dd, J = 15.5, 8.5 Hz), 1H), 2.75-2.68 (m, 3H), 1.96-1.92 (m, 2H); **^{13}C NMR** ($CDCl_3$, 100 MHz) δ 141.7, 128.4, 128.3, 127.2, 125.9, 124.6, 118.5, 109.1, 59.5, 38.4, 36.1, 32.9, 14.2. **HRMS** (+ESI) calculated for $C_{16}H_{18}N$ 224.14338, found 224.14303 $[M+H]^+$.

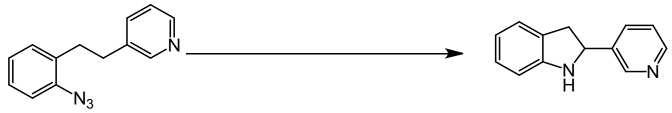

2-(pyridin-3-yl)indoline. Prepared by general procedure C using 3-(2-azidophenethyl)pyridine (150 mg, 0.669 mmol) and **$(Et_4N)_2[Co_2(L^{iPr})_2]$**[1] (7.0 mg, 0.007 mmol) in 5.0 mL of a 4:1 mixture of toluene and DMF, respectively. Purification by flash chromatography (7:3 EtOAc/hexanes) afforded the title compound as a light yellow oil (105 mg, 80 % yield). **R_f** (7:3 EtOAc/hexanes) 0.25; **IR** (thin film, cm^{-1}) 3677, 2851, 2370, 2277, 2037, 1483, 1249, 901, 753; **^{1}H NMR** ($CDCl_3$, 400 MHz) δ 8.63 (bs, 1H), 8.52 (d, J = 8.5 Hz, 1H), 7.79 (d, J = 7.9 Hz, 1H), 7.27-7.24 (m, 1H), 7.07 (dd, J = 15.4, 13.6, 2H), 6.74 (t, J = 7.2 Hz, 1H), 6.67 (d, J = 7.5 Hz, 1H), 4.97 (t, J = 9.2 Hz, 1H), 4.14 (bs, 1H), 3.46 (dd, J = 15.4, 9.2 Hz, 1H), 2.95 (dd, J = 15.8, 9.2 Hz, 1H); **^{13}C NMR** ($CDCl_3$, 100 MHz) δ 150.4, 148.0, 147.3, 134.9, 127.8, 127.4, 124.6, 123.9, 119.4, 109.2, 60.9, 39.5, 29.7; **HRMS** (+ESI) calculated for $C_{13}H_{13}N_2$ 197.1073, found 197.1074 $[M+H]^+$.

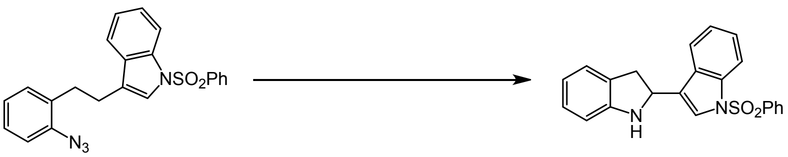

3-(indolin-2-yl)-1-(phenylsulfonyl)-1H-indole. Prepared by general procedure C using 3-(2-azidophenethyl)-1-(phenylsulfonyl)-1H-indole (150 mg, 0.373 mmol) and **$(Et_4N)_2[Co_2(L^{iPr})_2]$**[1] (4.0 mg, 0.004 mmol) in 5.0 mL of a 4:1 mixture of toluene and DMF, respectively. Purification by flash chromatography (7:1 hexanes/EtOAc) afforded the title compound as a yellow oil (120 mg, 85 % yield). **R_f** 0.18 (7:1 hexanes/EtOAc); **IR** (thin film, cm^{-1}) 3369, 3030, 1608, 1484, 1246, 1033, 747; **^{1}H NMR** ($CDCl_3$, 400 MHz) δ 8.01 (d, *J* = 8.4, 1.1 Hz, 1H), 7.91 - 7.84 (m, 2H), 7.57 (s, 1H), 7.55 - 7.48 (m, 2H), 7.47 - 7.38 (m, 2H), 7.36 - 7.27 (m, 1H), 7.26 - 7.17 (m, 1H), 7.16 - 7.03 (m, 2H), 6.76 (td, *J* = 7.4, 1.1 Hz, 1H), 6.69 (d, *J* = 7.7 Hz, 1H), 5.16 (tdd, *J* = 9.2, 3.3, 0.9 Hz, 1H), 4.12 (s, 1H), 3.48 (dd, *J* = 15.6, 9.3 Hz, 1H), 3.14-3.03 (m, 1H); **^{13}C NMR** ($CDCl_3$, 100 MHz) δ 150.5, 138.1, 135.8, 133.8, 129.3, 129.0, 128.1, 127.7, 126.8, 125.8, 124.9, 124.7, 123.2, 122.7, 120.2, 119.1, 113.9, 109.3, 56.0, 37.3; **HRMS** (+ESI) calculated for $C_{22}H_{19}N_2O_2S$ 375.1167, found 375.1162 $[M+H]^+$.

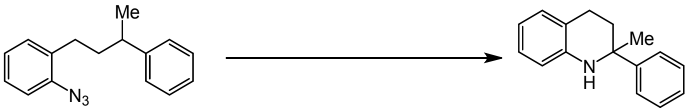

2-methyl-2-phenyl-1,2,3,4-tetrahydroquinoline. Prepared by general procedure C using 1-azido-2-(3-phenylbutyl)benzene (150 mg, 0.597 mmol) and **$(Et_4N)_2[Co_2(L^{iPr})_2]$**[1] (6.3 mg, 0.006 mmol) in 5.0 mL of a 4:1 mixture of toluene and DMF, respectively. Purification by flash chromatography (9:1 hexanes/EtOAc) afforded the title compound as a yellow oil (120 mg, 85 % yield). **R_f** 0.8 (9:1 hexanes/EtOAc); **IR** (thin film, cm^{-1}) 3402, 3052, 3023, 2924, 2843, 2358, 2345; **^{1}H NMR** ($CDCl_3$, 400 MHz) δ 7.38 (dd, *J* = 8.8, 1.2 Hz, 2H), 7.28 (t, *J* = 7.9 Hz, 2H), 7.19 (td, *J* = 7.6, 1.2 Hz, 1H), 7.02 (t, *J* = 7.6 Hz, 1H), 6.9 (d, *J* = 7.6 Hz, 1H), 6.61-6.58 (m, 2H), 4.11 (bs, 1H), 2.59 (dt, *J* = 16.4, 4.7 Hz, 1H), 2.31 (ddd, *J* = 16.2, 11.3, 5.3 Hz, 1H), 2.20 (dt, *J* = 12.9, 5.0 Hz, 1H) 1.9 (ddd, *J* = 12.9, 11.1, 4.7 Hz, 1H), 1.57 (s, 3H); **^{13}C NMR** ($CDCl_3$, 100 MHz) δ 148.2, 143.8, 129.2, 128.3, 126.9, 126.3, 125.4, 120.4, 116.6, 113.4, 55.5, 35.4, 30.6, 24.3; **HRMS** (+ESI) calculated for $C_{16}H_{18}N$ 224.1434, found 224.1436 $[M+H]^+$.

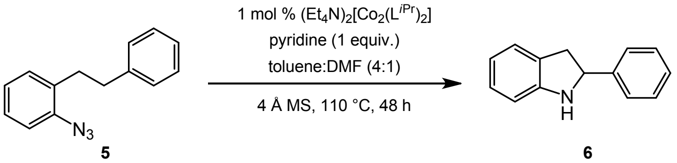

5 → **6** 78 %

2-phenylindoline (6). General procedure C was modified as follows: to a mixture of azide **5**[2] (170 mg, 0.761 mmol) and **$(Et_4N)_2[Co_2(L^{iPr})_2]$**[1] (8.0 mg, 0.0076 mmol) was added 0.06 mL of pyridine (0.761 mmol) in 5.0 mL of a 4:1 mixture of toluene and DMF, respectively. Purification by flash chromatography (7:1 hexanes/EtOAc) afforded the title compound (133 mg, 78 % yield).[2]

e) Control reactions. Several control reactions were conducted to ensure the cobalt catalysts were required for amination. Heating azide **5** at 110 °C for 48 hours in the presence of molecular sieves in a toluene:DMF (4:1) mixture did not lead to the formation of indoline **6;** starting material was isolated in greater than 95% yield. Heating azide **5** at 110 °C for 48 hours in the presence of molecular sieves and $CoBr_2$ in a toluene:DMF (4:1) mixture did not lead to the formation of indoline **6;** starting material was isolated in greater than 95% yield. Similarly, azide **5** does not react under similar conditions in the presence of free ligand.

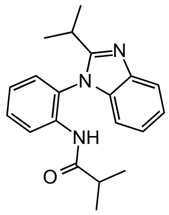

f) Cyclized Ligand Byproduct*: N*-(2-(2-isopropyl-1H-benzo[d]imidazol-1-yl)phenyl)isobutyramide
This cyclized ligand byproduct of catalyst **1** is observed when the amination reactions are run in wet organic solvents and/or in the absence of activated molecular sieves. Under these conditions, we see diminished indoline yields and observe and isolate this cyclized ligand product. This cyclized ligand species was isolated by column chromatography. **^{1}H NMR** ($CDCl_3$, 400 MHz) δ 8.51 (d, *J* = 8.4 Hz, 1H), 7.87 (d, *J* = 8.0 Hz, 1H), 7.56 (m, 1H), 7.32 (t, *J* = 7.6 Hz, 1H), 7.31 – 7.27 (m, 2H), 7.22, (t, *J* = 7.6 Hz, 1H), 6.95 (8, *J* = 7.6 Hz, 1H), 6.53 (bs, 1H), 2.93 (septet, *J* = 6.8 Hz 1H), 2.12 (septet, *J* = 6.8 Hz, 1H), 1.37 (d, *J* = 7.2, 3H), 1.27 (d, *J* = 7.2, 3H), 0.88 (d, *J* = 6.8, 3H), 0.87 (d, *J* = 6.8, 3H); **HRMS** (+ESI) calculated for $C_{20}H_{24}N_3O$ 322.1919, found 322.1903 $[M+H]^+$.

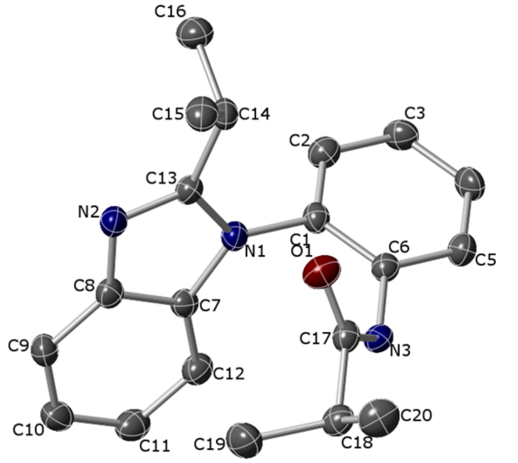

Figure S1. Thermal ellipsoid diagram of *N*-(2-(2-isopropyl-1H-benzo[d]imidazol-1-yl)phenyl)isobutyramide drawn at 50% probability. Hydrogen atoms have been removed for clarity. Selected bond lengths (Å): O1–C17 1.220(3), N2–C13 1.320(3), N2–C8 1.396(3), N3–C6 1.415(3), N3–C17 1.360(3), N1–C1 1.430(3), N1–C7 1.386(3), N1–C13 1.383(3).

Table S1. X-ray crystallographic data for *N*-(2-(2-isopropyl-1H-benzo[d]imidazol-1-yl)phenyl)isobutyramide

**Formula**	$C_{40}H_{46}N_6O_2$
**Form. Wt. (g/mol)**	642.83
**T (K)**	173(2)
**Crystal system**	monoclinic
**Space group**	$P2_1/c$
a **(Å)**	8.8762(18)
b **(Å)**	13.892(3)
c **(Å)**	14.471(3)
**α (°)**	90
**β (°)**	100.77(3)
**γ (°)**	90
**V (Å^3)**	1753.0(6)
**Z**	2
**ρ, calcd (mg/mm^3)**	1.218
**m/mm^{-1}**	0.077
**Crystal size (mm^3)**	0.188 × 0.137 × 0.064
**Theta range**	4.1 to 58.5°
**Reflns. Collected**	32111
**Unique reflns.**	4760
**Data/rest./par.**	4760/0/221
GOF	0.970
**Final R indexes [I>2σ (I)]**	R_1 = 0.0606, wR_2 = 0.1179
**All data**	R_1 = 0.1580, wR_2 = 0.1546

III. Kinetic Isotope Effect Experiment

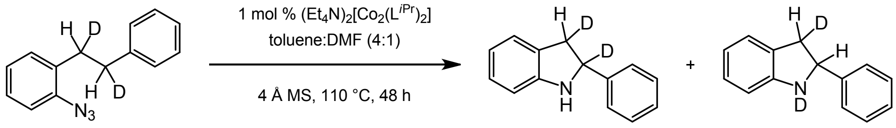

7 **8-H** **8-D**

$k_H/k_D = 3.4$

Prepared by general procedure C azide **7**[7] (150 mg, 0.666 mmol) and **$(Et_4N)_2[Co_2(L^{iPr})_2]$**[1] (7.0 mg, 0.0067 mmol) in 5.0 mL of a 4:1 mixture of toluene and DMF, respectively. Following filtration through Celite and removal of solvent *in vacuo*, ^{1}H NMR indicated formation of **8-D** and **8-H**. The mixture of indolines **8-D** and **8-H** were purified by flash chromatography. The kinetic isotope effect (KIE) was determined from the ratio of **8-D** and **8-H**, which were found identical in both the crude reaction mixture and purified product mixture.

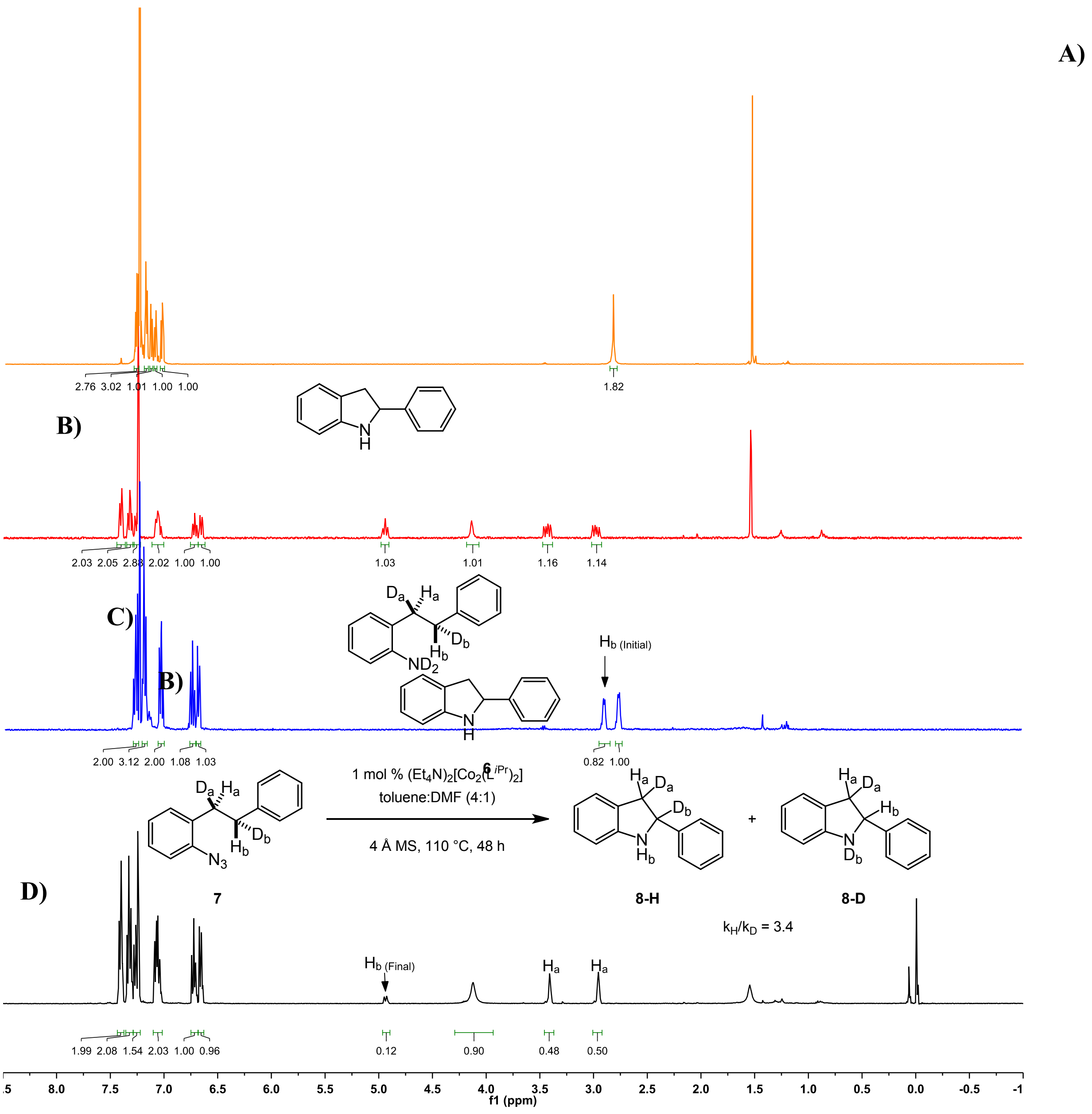

Figure S-2. ^{1}H NMR spectra for **A)** deuterated azide **7**, **B)** indoline product **6**, **C)** aniline precursor to azide **7**,[7] and **D)** reaction mixture of indoline products **8-D** and **8-H**.

Calculations used for the determination of KIE value from ^{1}H NMR analysis found in Figure S-2:

Before Reaction (H_b initial): 0.82 H_b

After Reaction (H_b final): 0.12 H_b

H_b consumed : (H_b initial) - (H_b final) = (0.82 - 0.12) = 0.70

% of H_b consumed = (0.70/0.82) x 100% = 85%

Before Reaction (D_b initial): 2.0 - (H_b initial) = 2.0 - 0.82 = 1.18 D_b

After Reaction (D_b final): 1 - (H_b initial) = 1.0 - 0.12 = 0.88

D_b consumed : (D_b initial) - (D_b final) = (1.18 - 0.88) = 0.30

% of D_b consumed = (0.30 / 1.18) x 100% = 25%

KIE = K_H / K_D = 0.85 / 0.25 = **3.4**

IV. ^{1}H and ^{13}C NMR Spectra

2,2'-Bis(*tert*-butylacetamido)diphenylamine

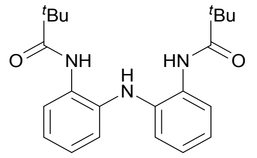

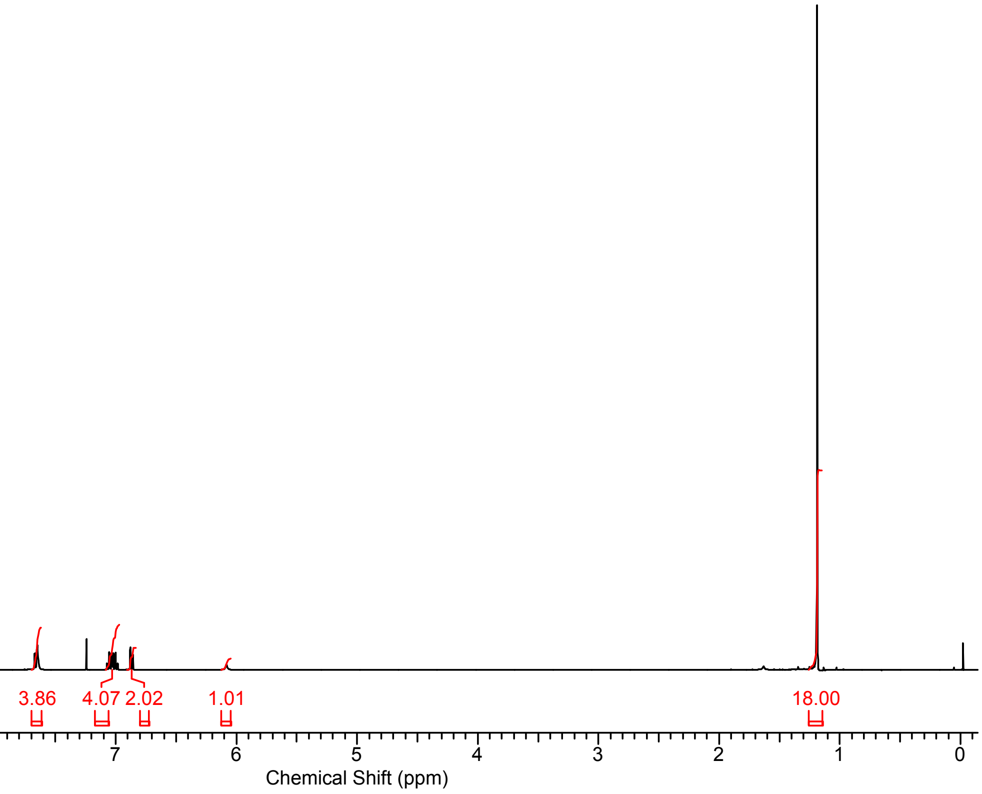

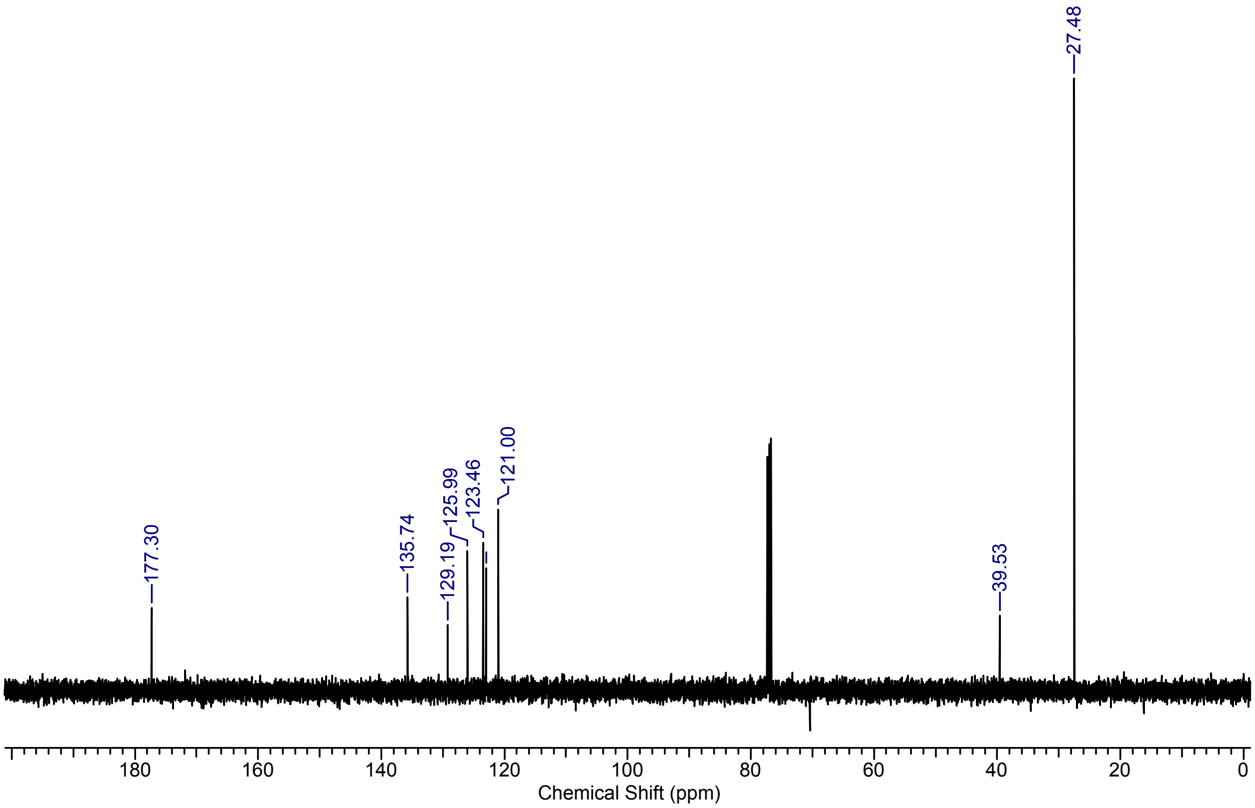

2,2'-Bis(diphenylacetamido)diphenylamine

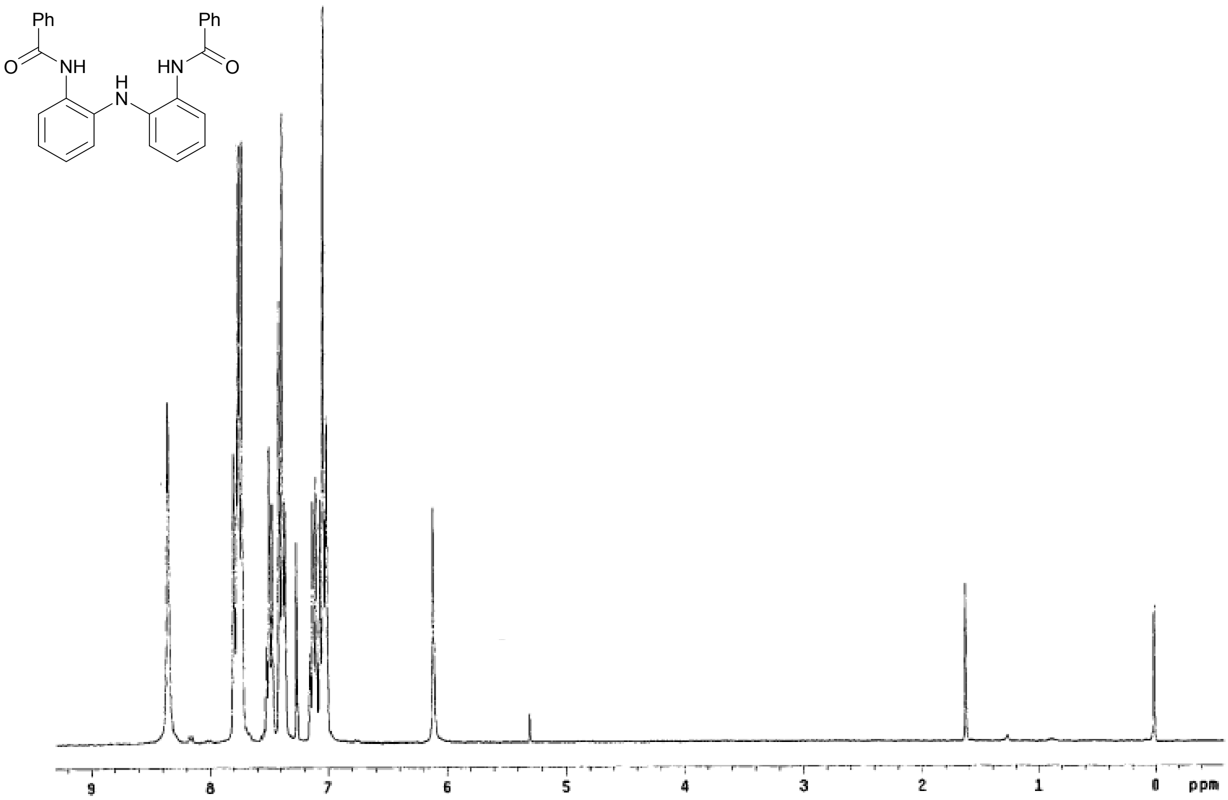

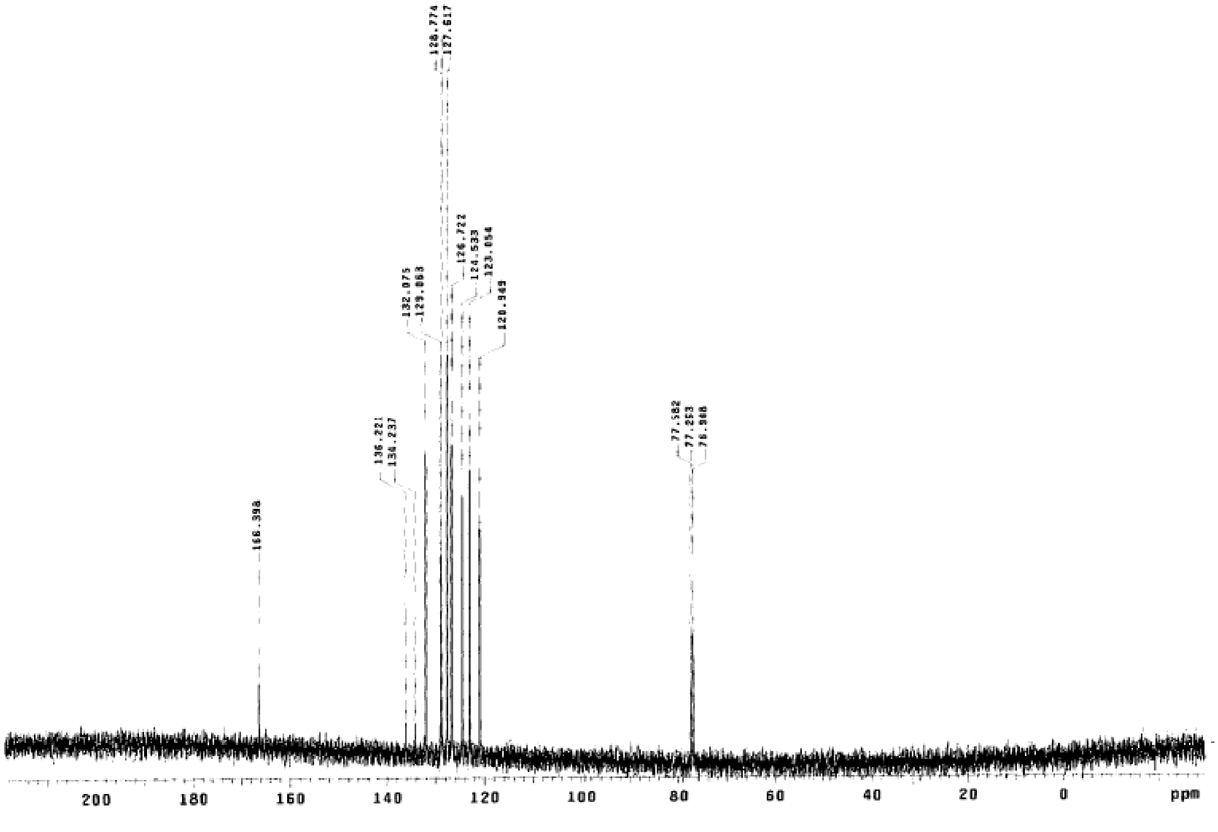

2,2'-Bis(trifluoroacetamido)diphenylamine

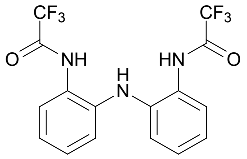

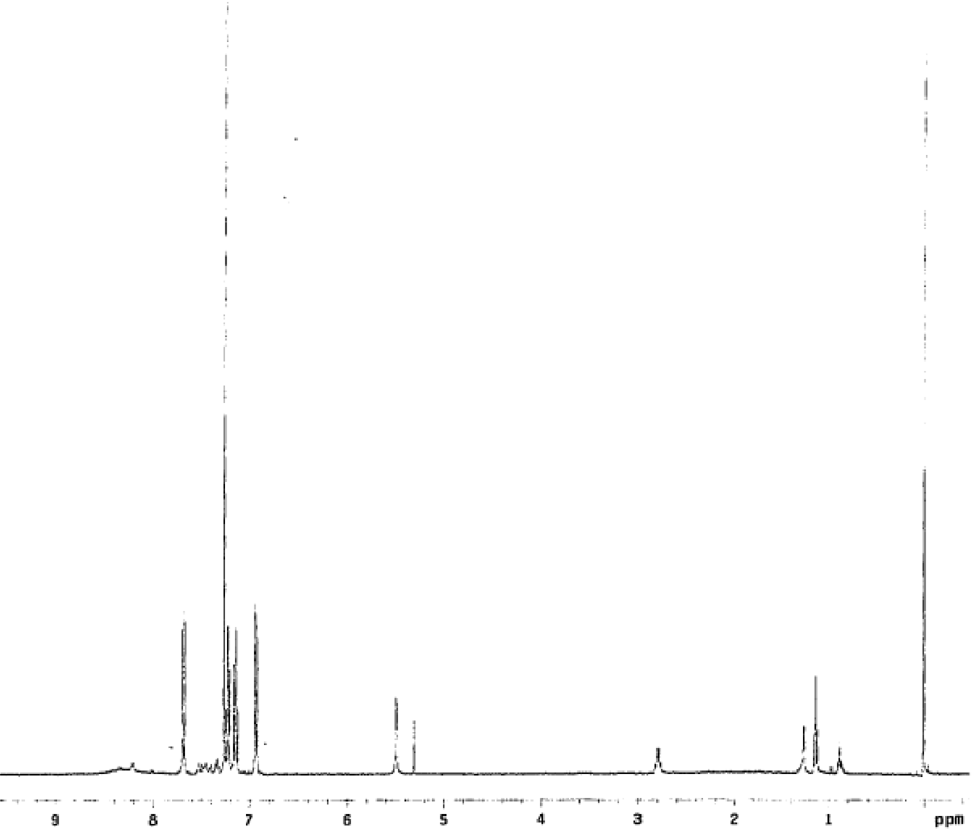

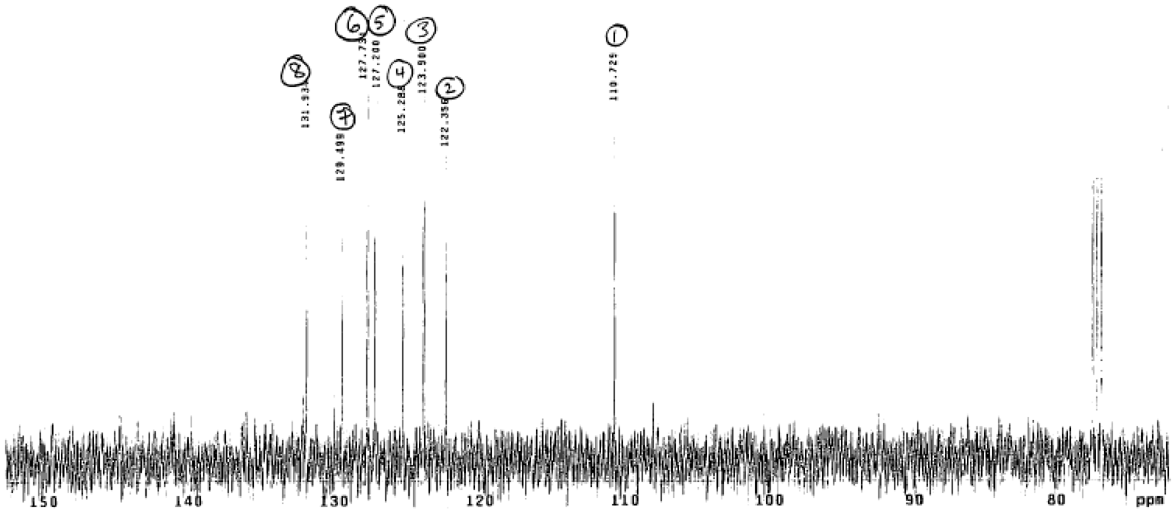

2-(3-phenylprop-1-yn-1-yl)aniline

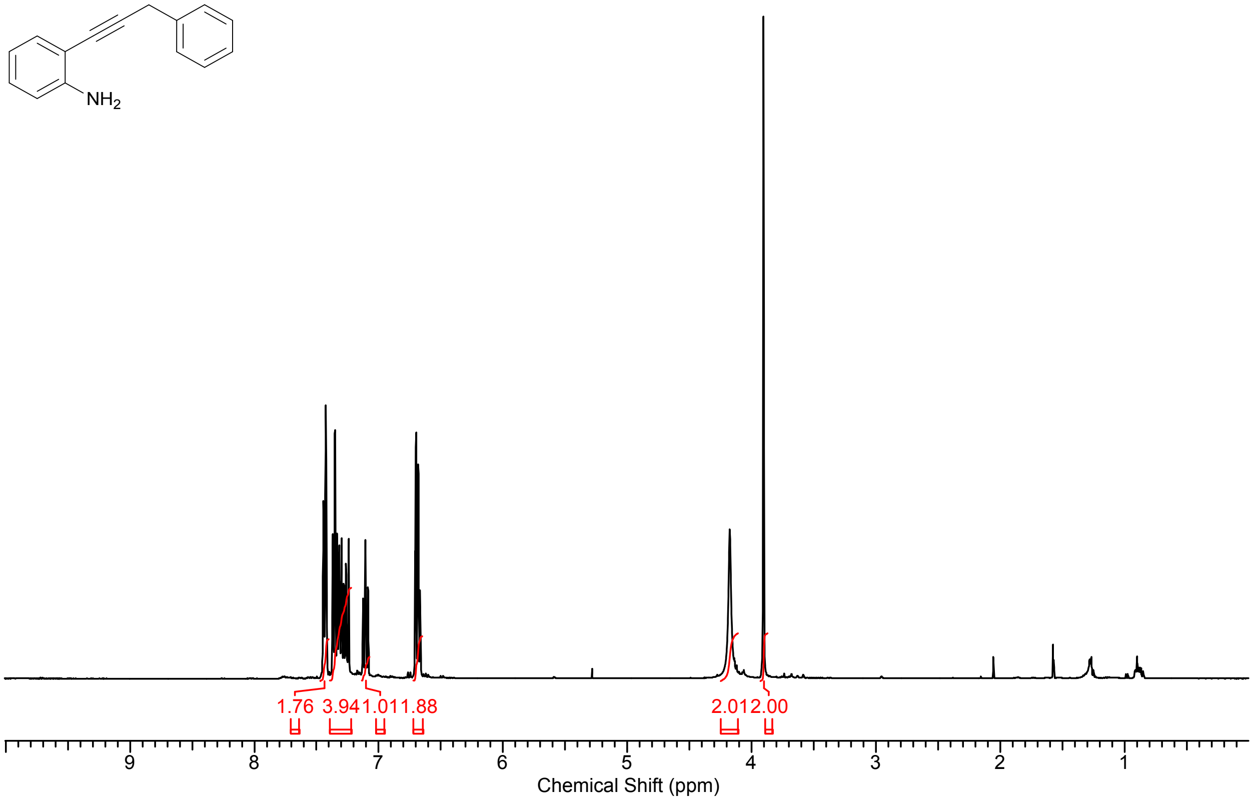

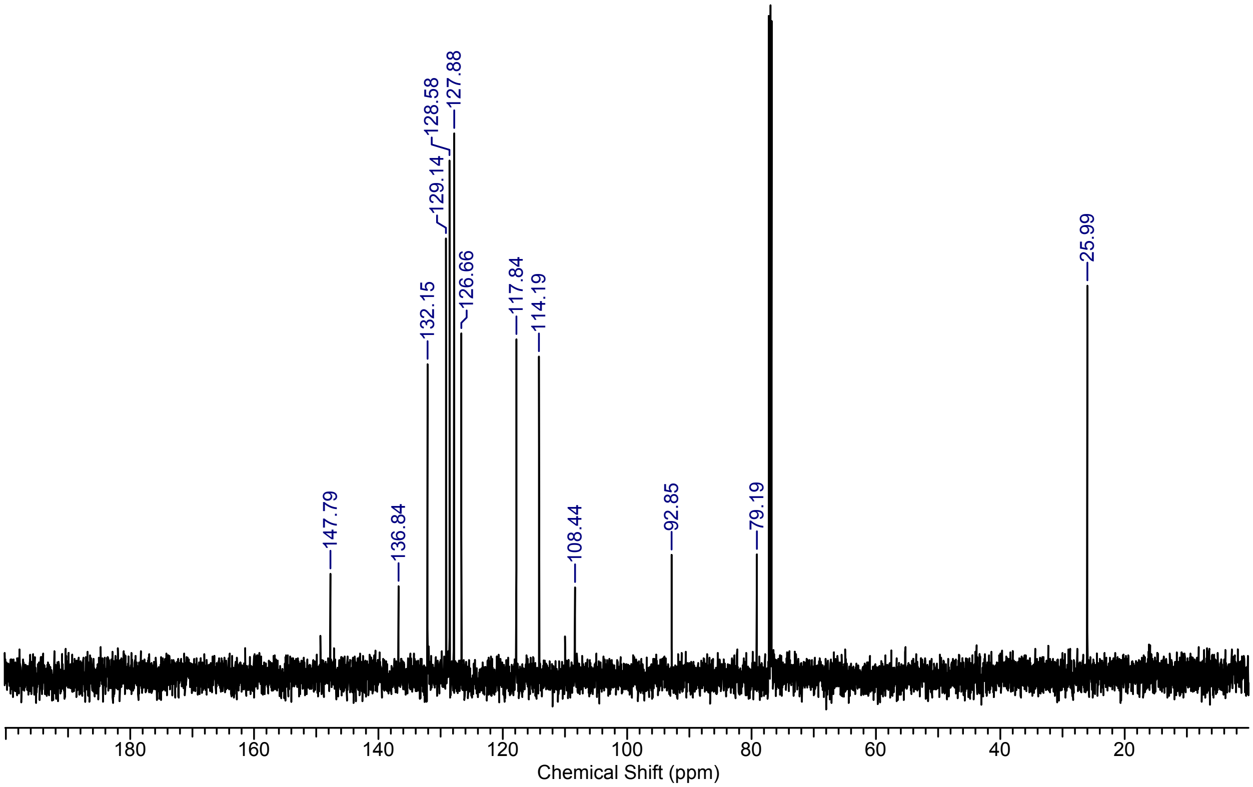

1-azido-2-(3-phenylpropyl)benzene

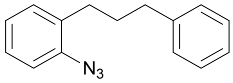

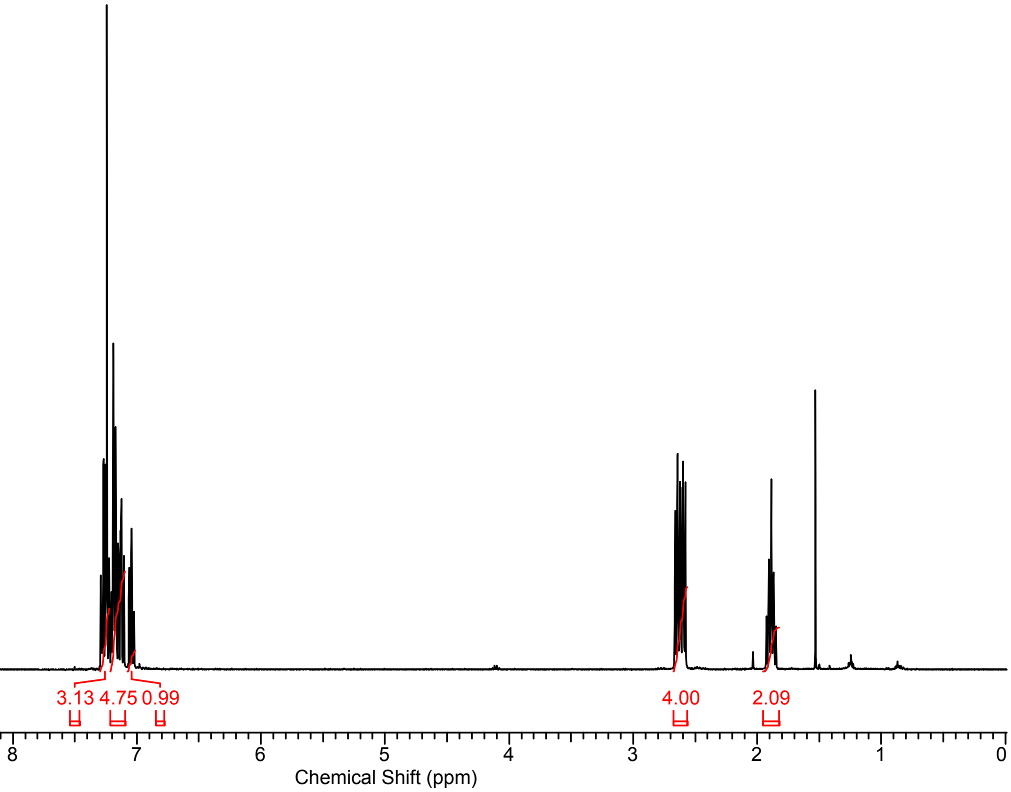

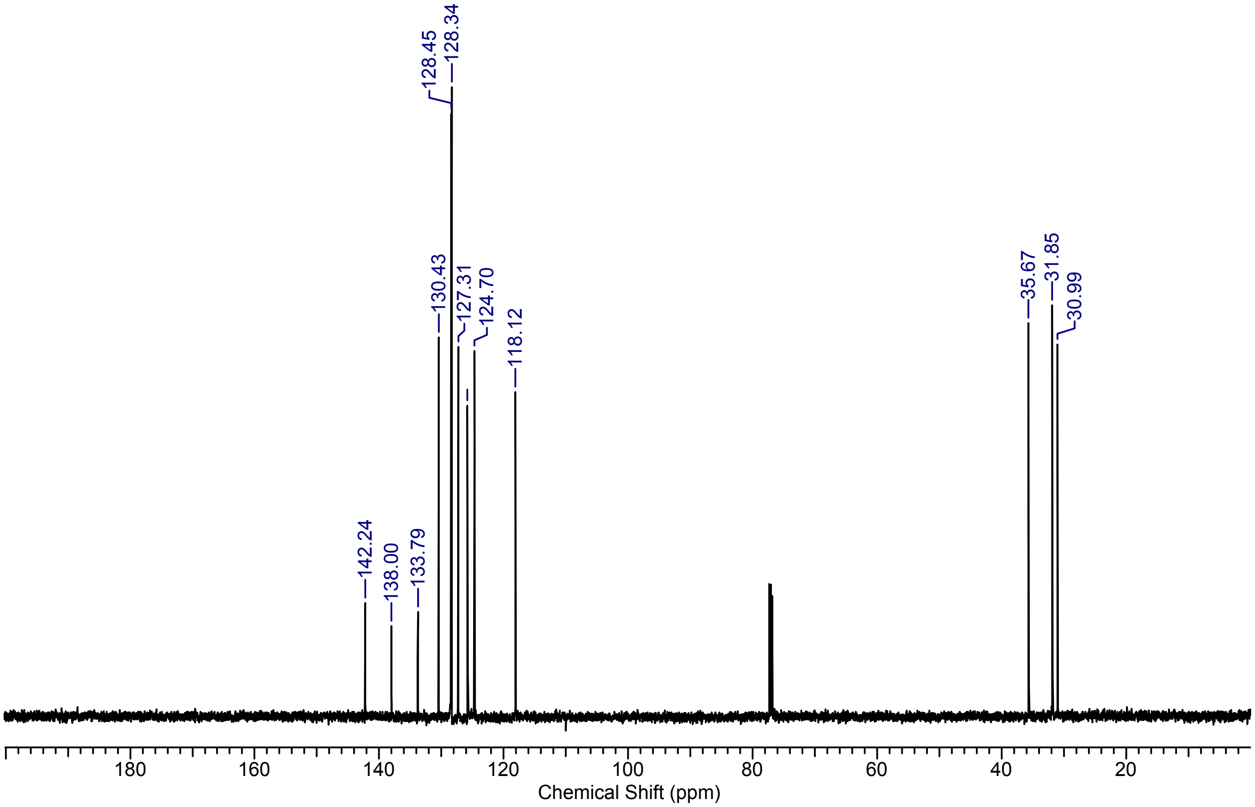

2-(4-phenylbut-1-yn-1-yl)aniline

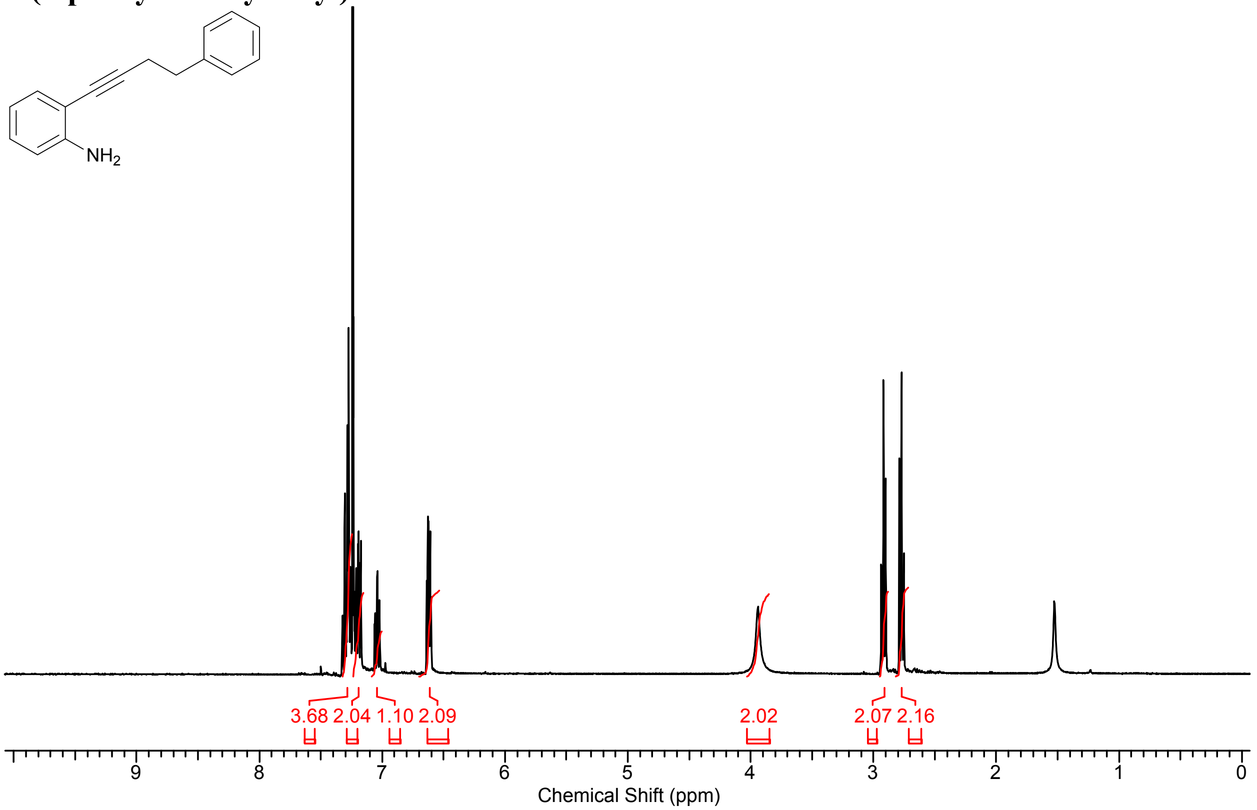

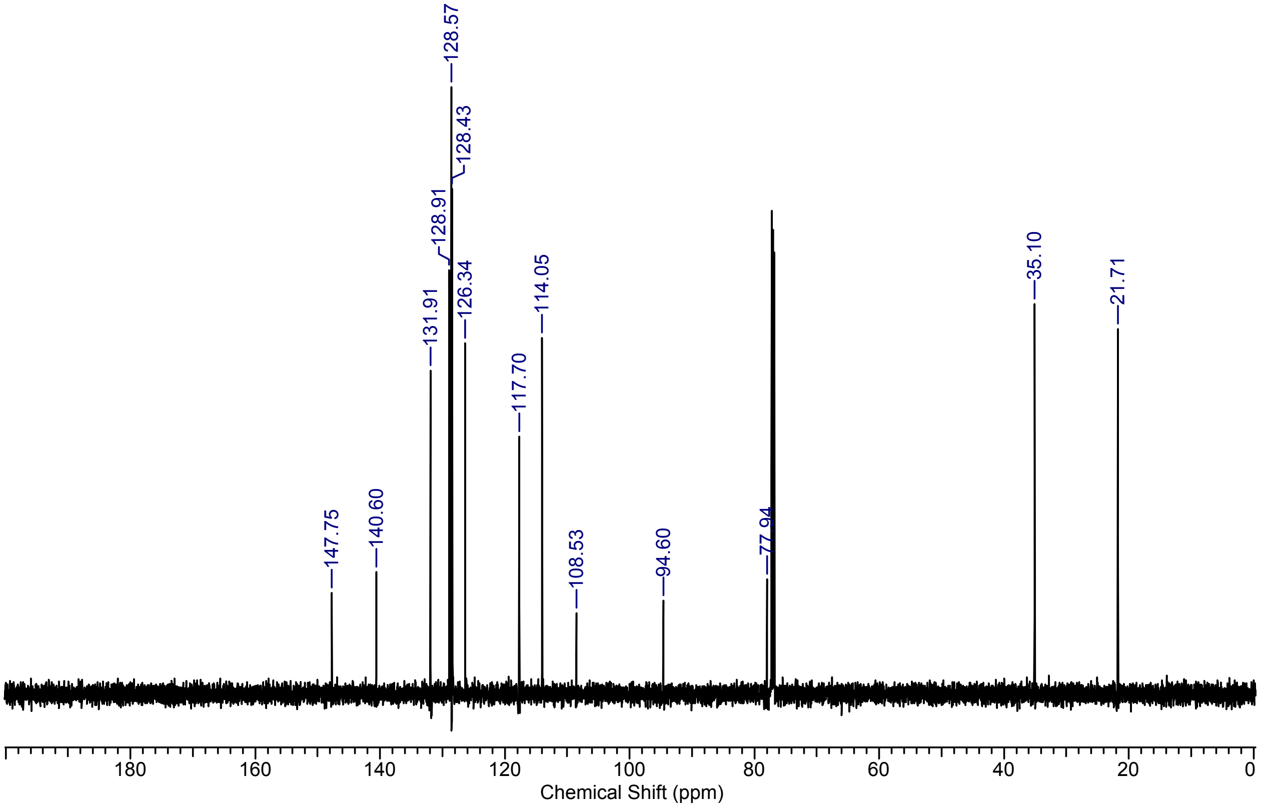

1-azido-2-(4-phenylbutyl)benzene

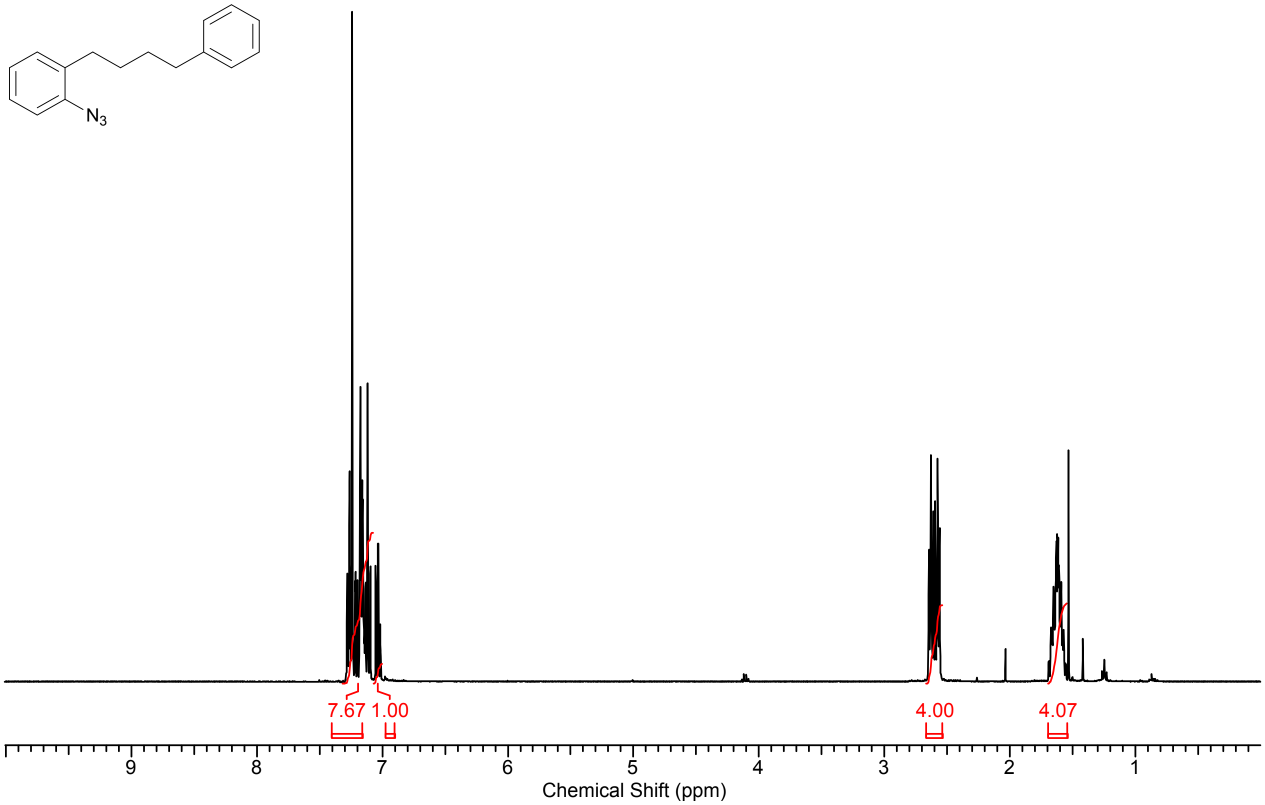

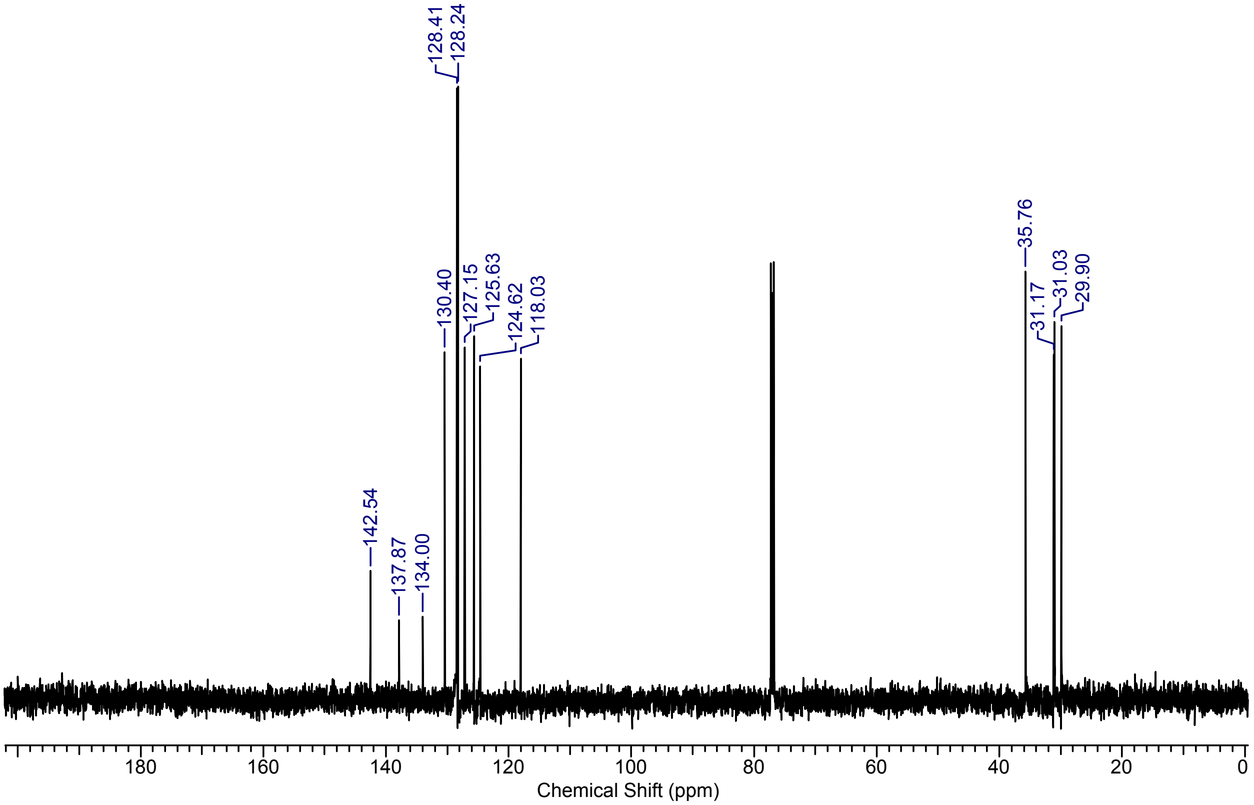

2-(pyridin-3-ylethynyl)aniline

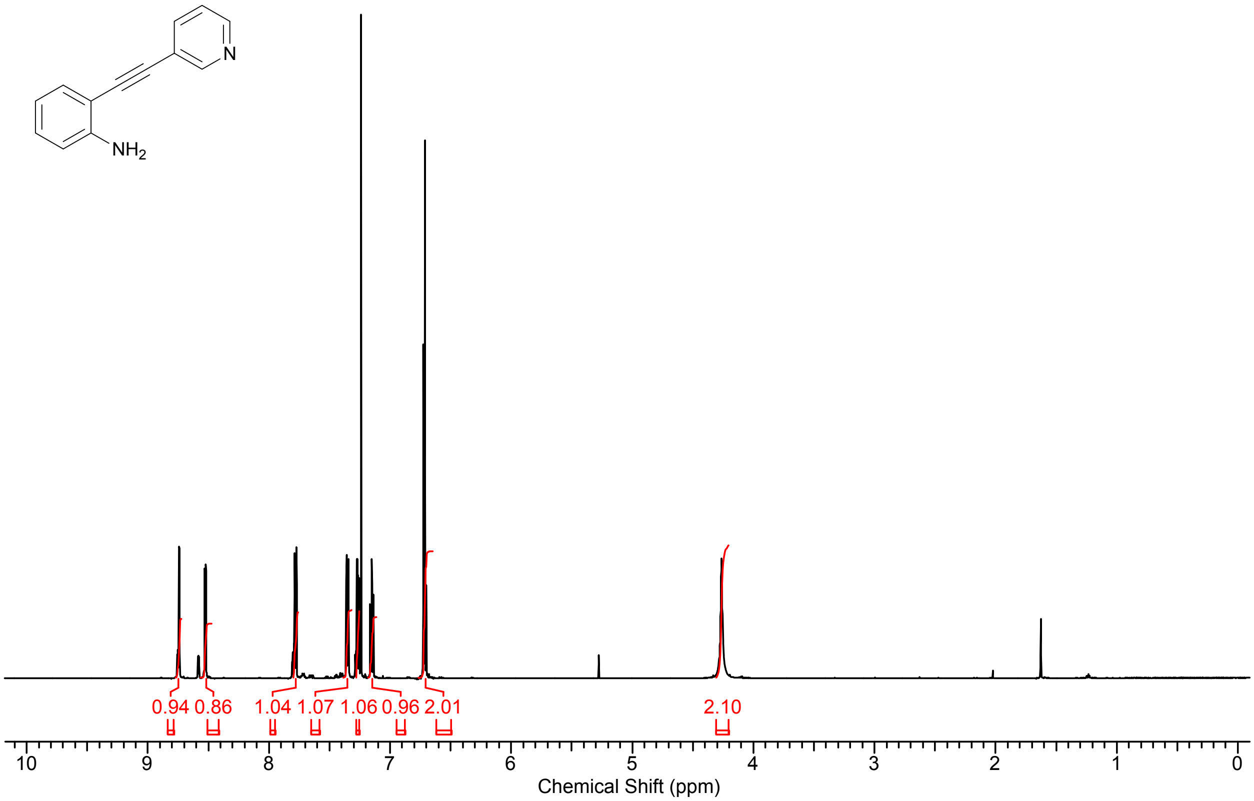

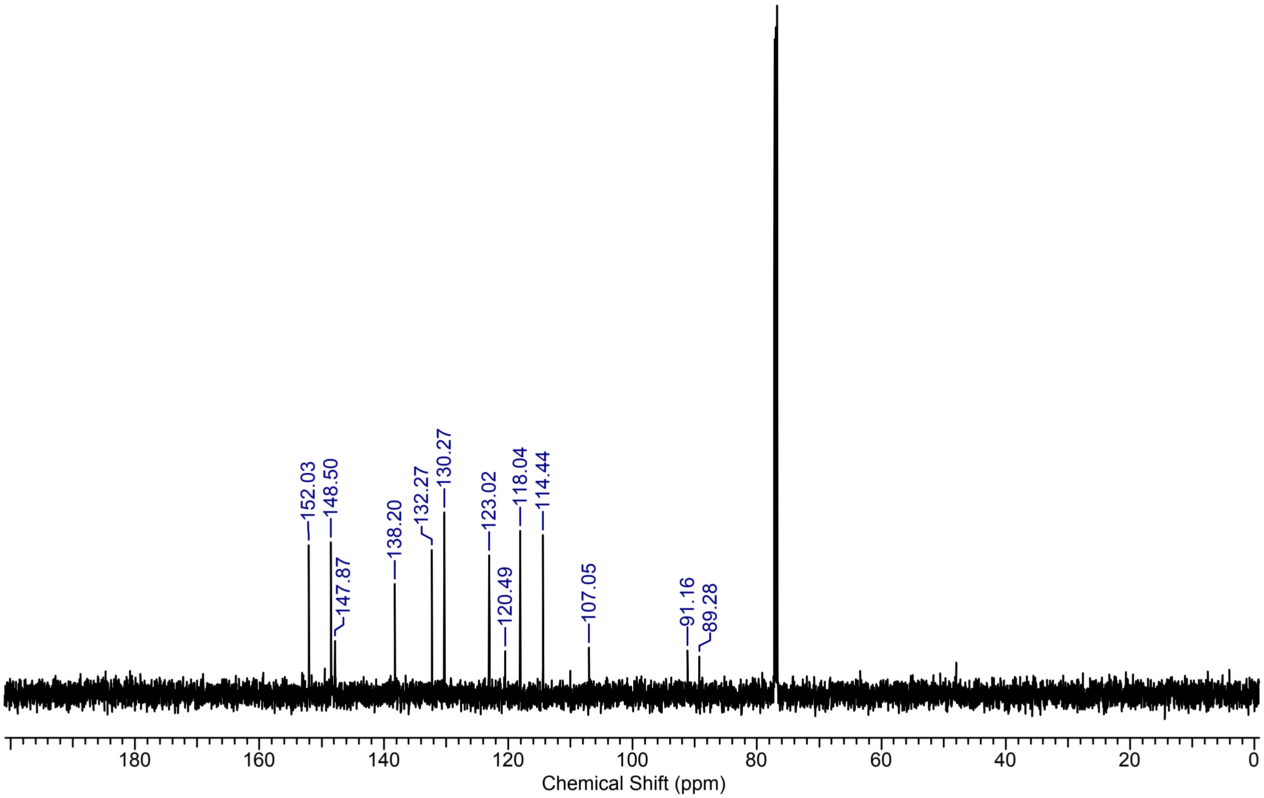

3-(2-azidophenethyl)pyridine

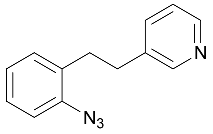

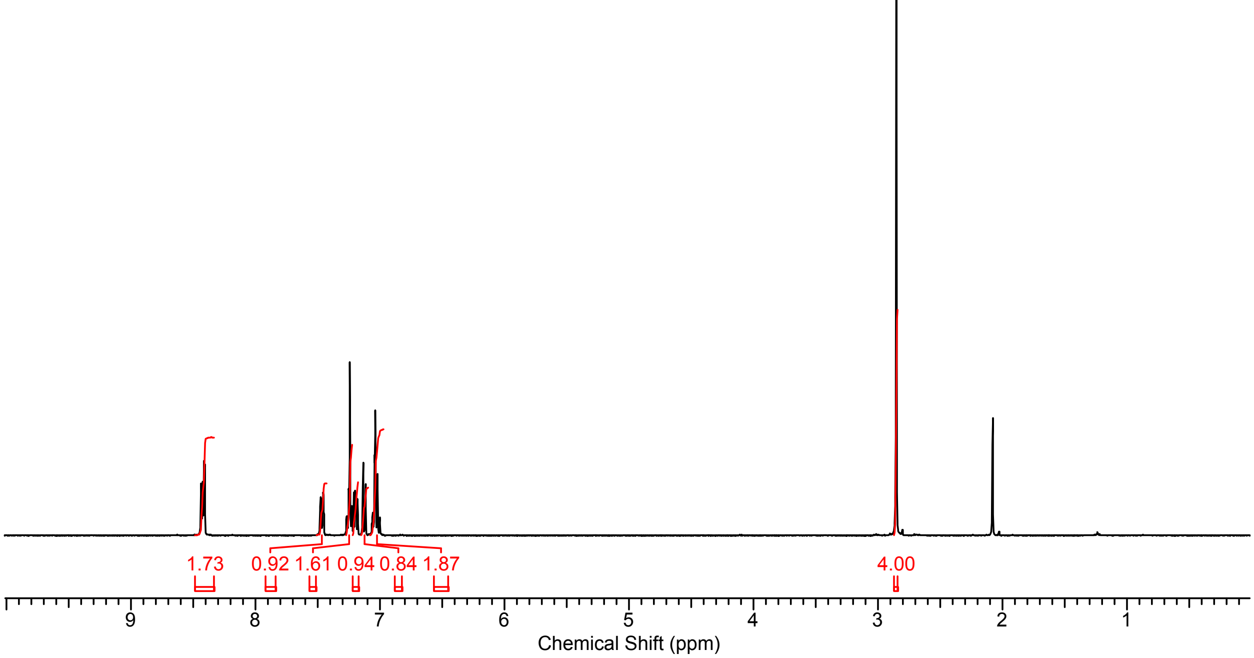

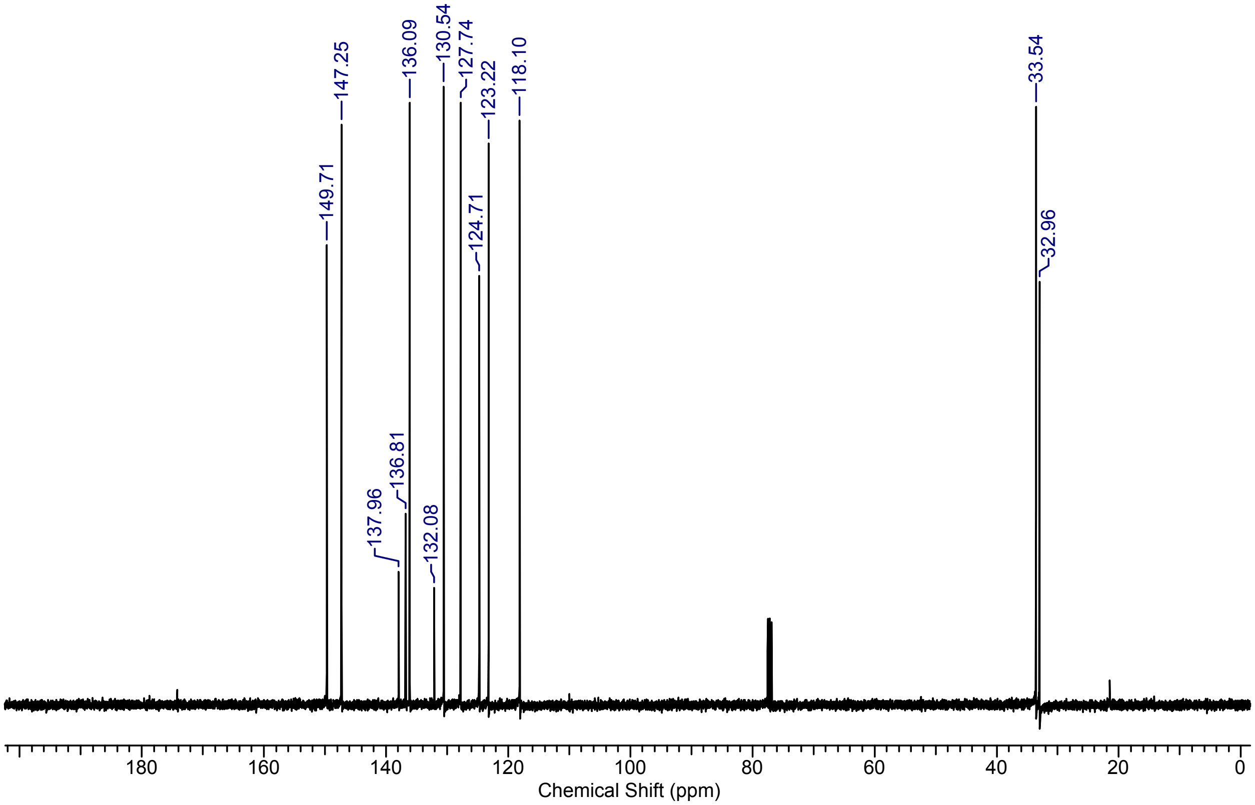

Phosphonium salt

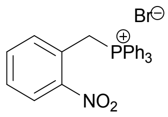

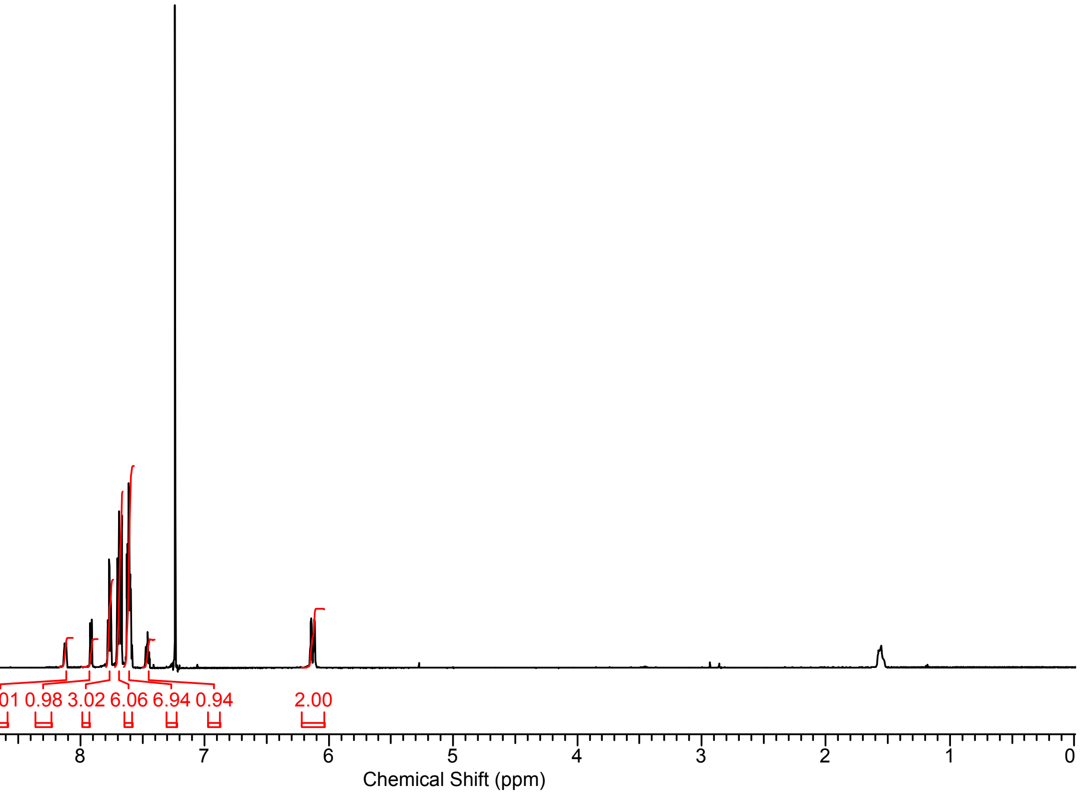

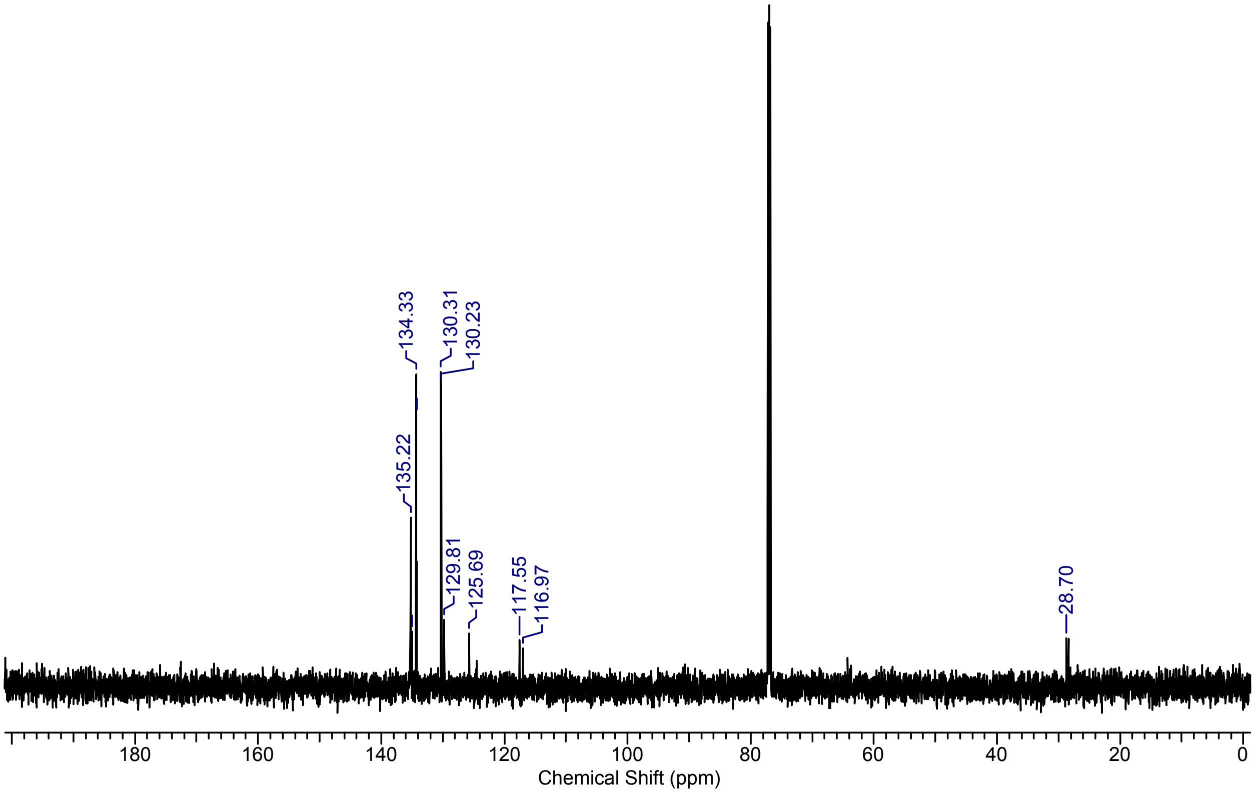

3-(2-azidophenethyl)-1-(phenylsulfonyl)-1H-indole

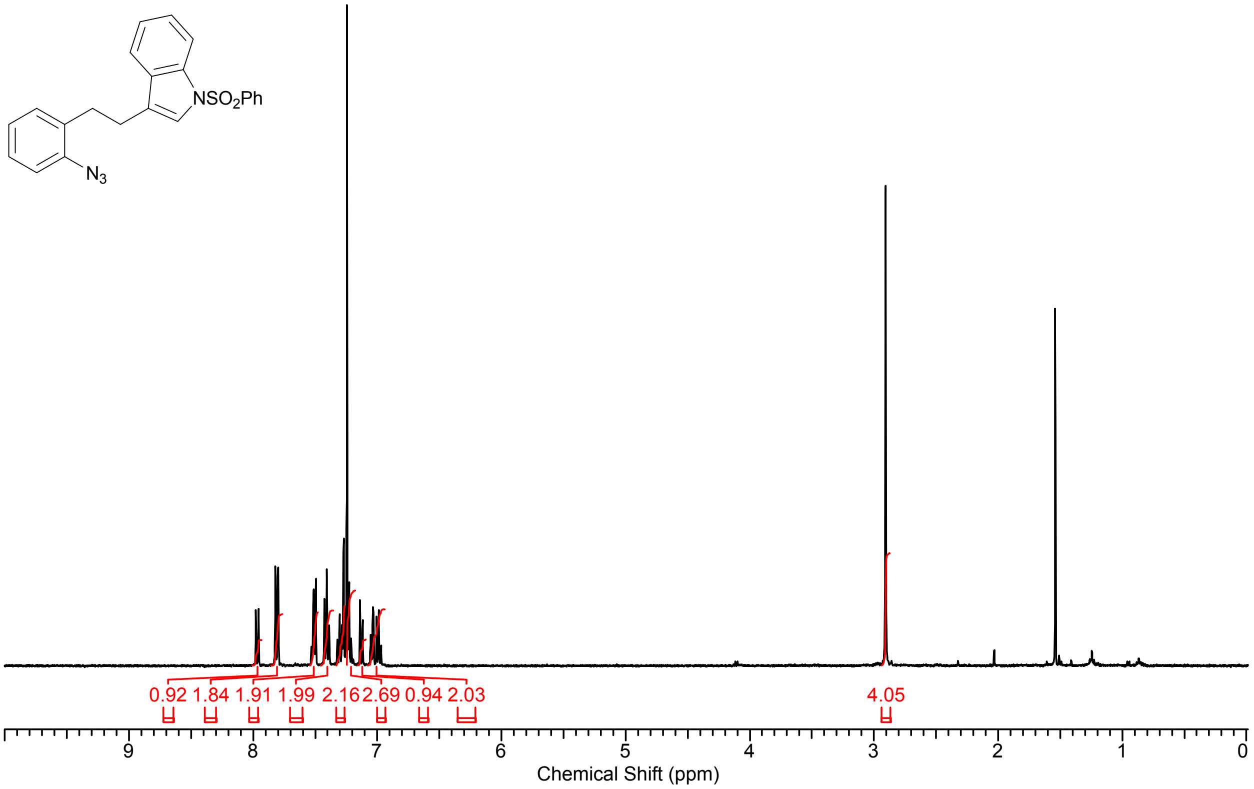

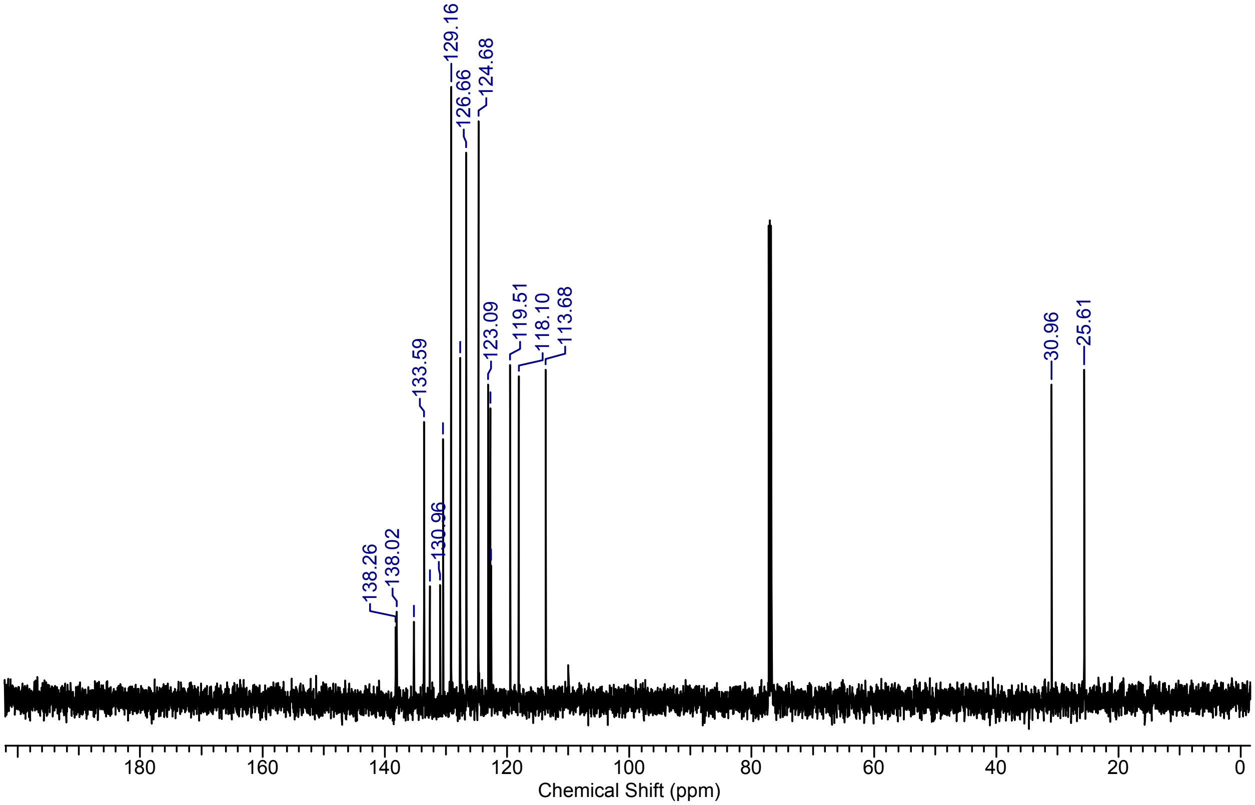

2-(3-phenylbut-1-yn-1-yl)aniline

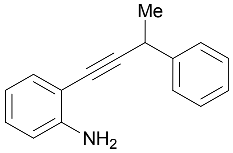

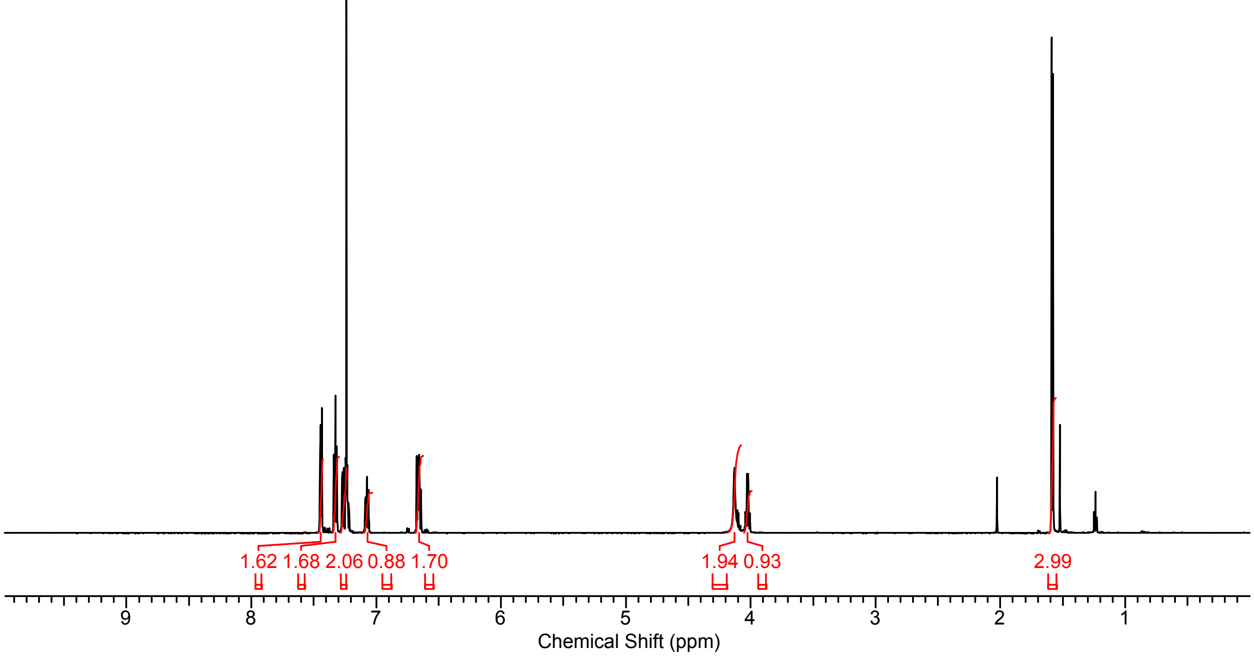

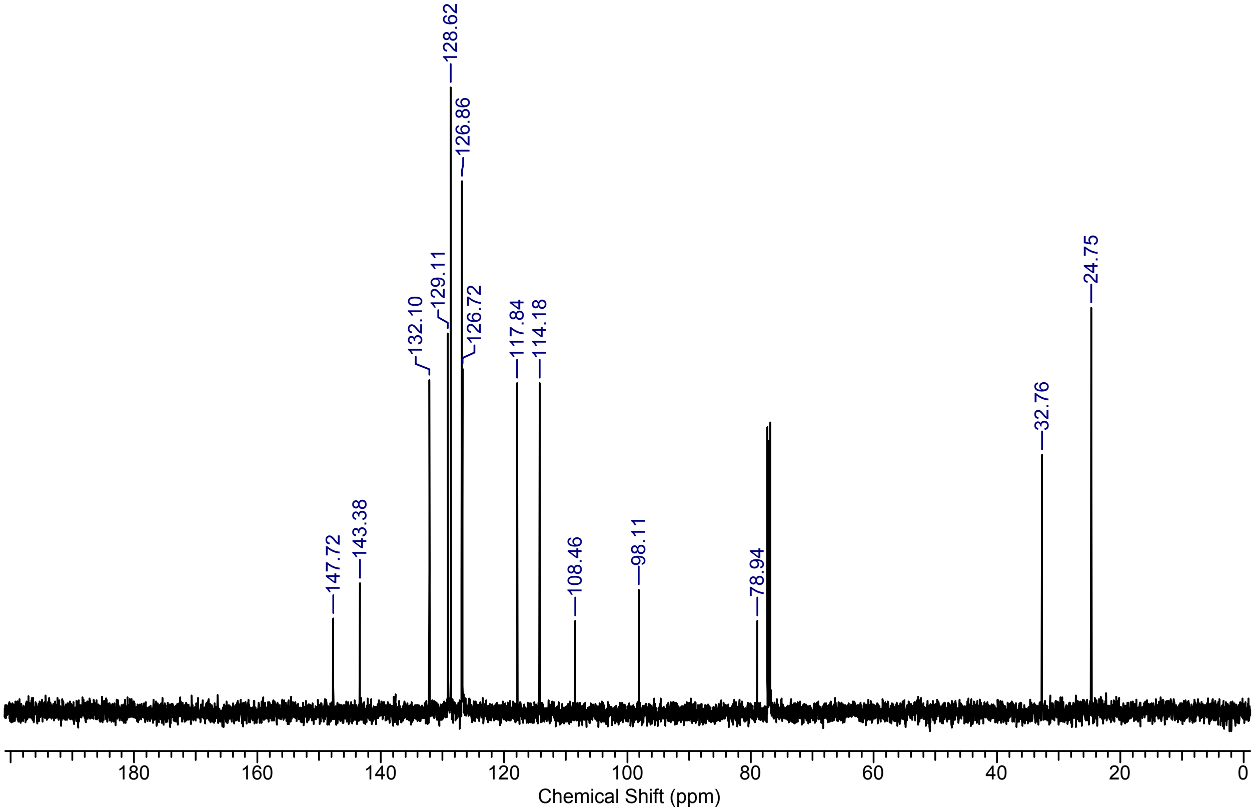

1-azido-2-(3-phenylpropyl)benzene

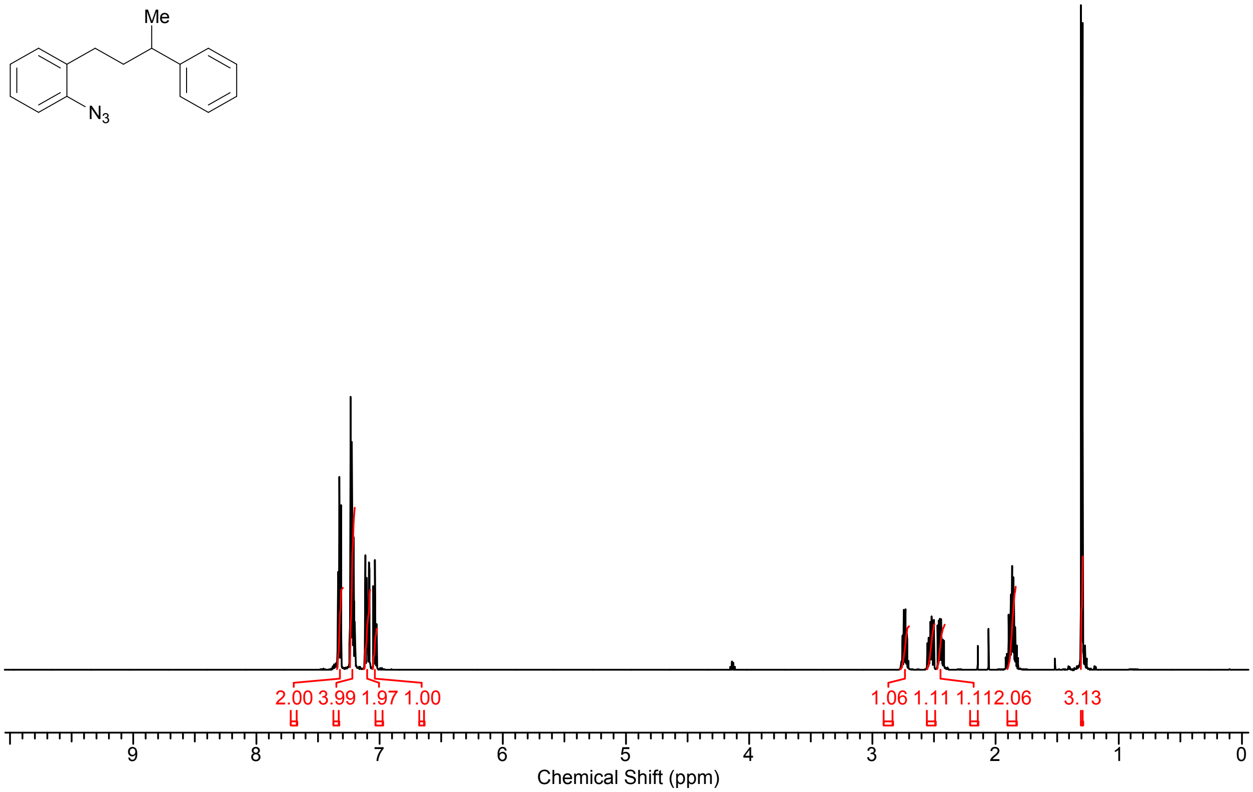

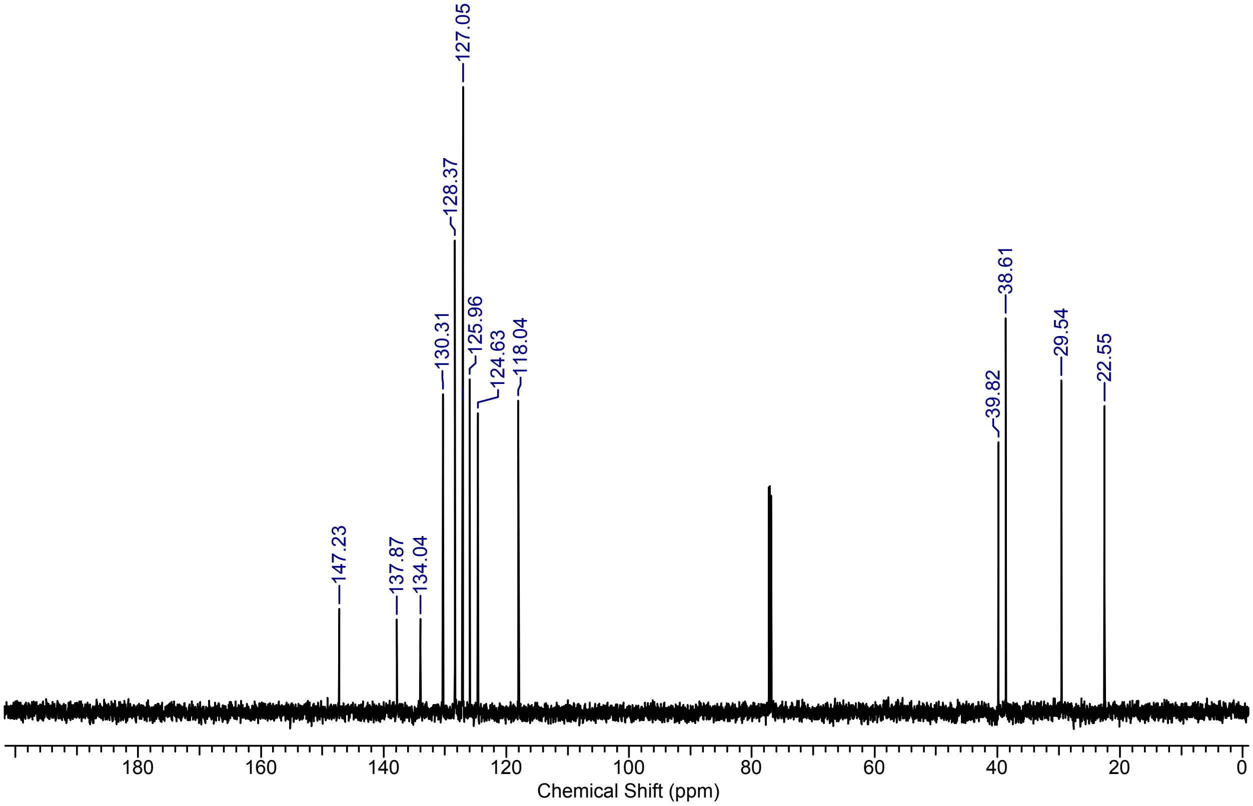

2-phenyl-2,3,4,5-tetrahydro-1H-benzo[b]azepine

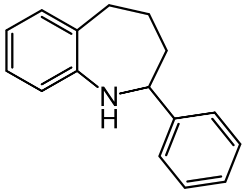

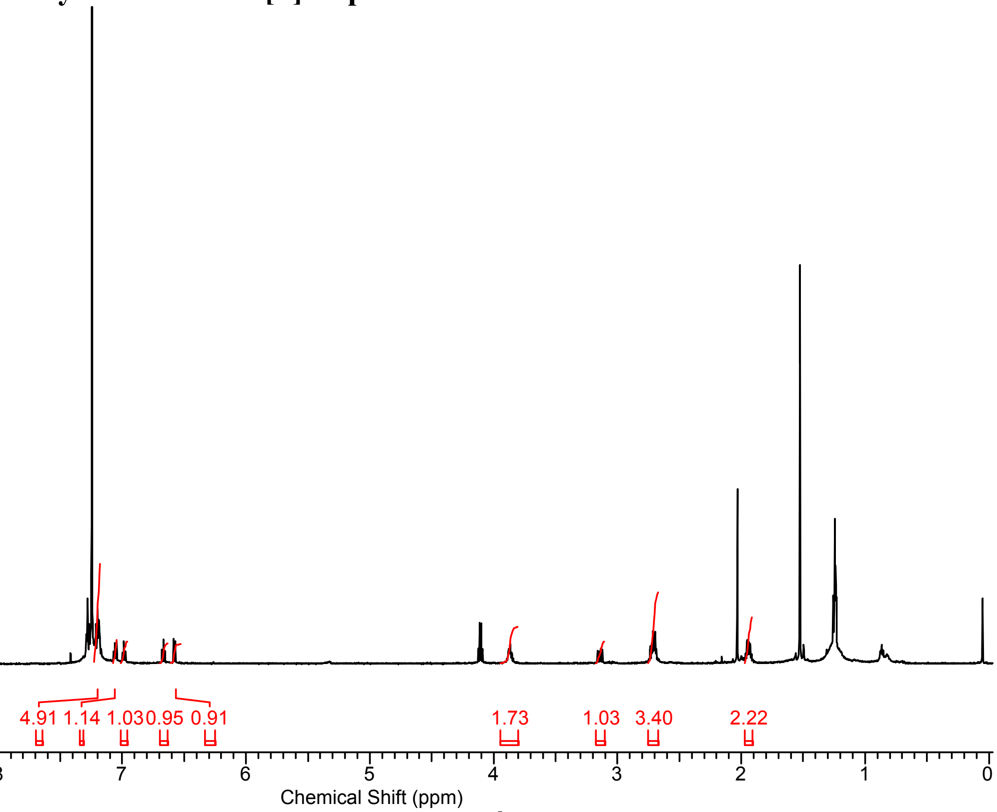

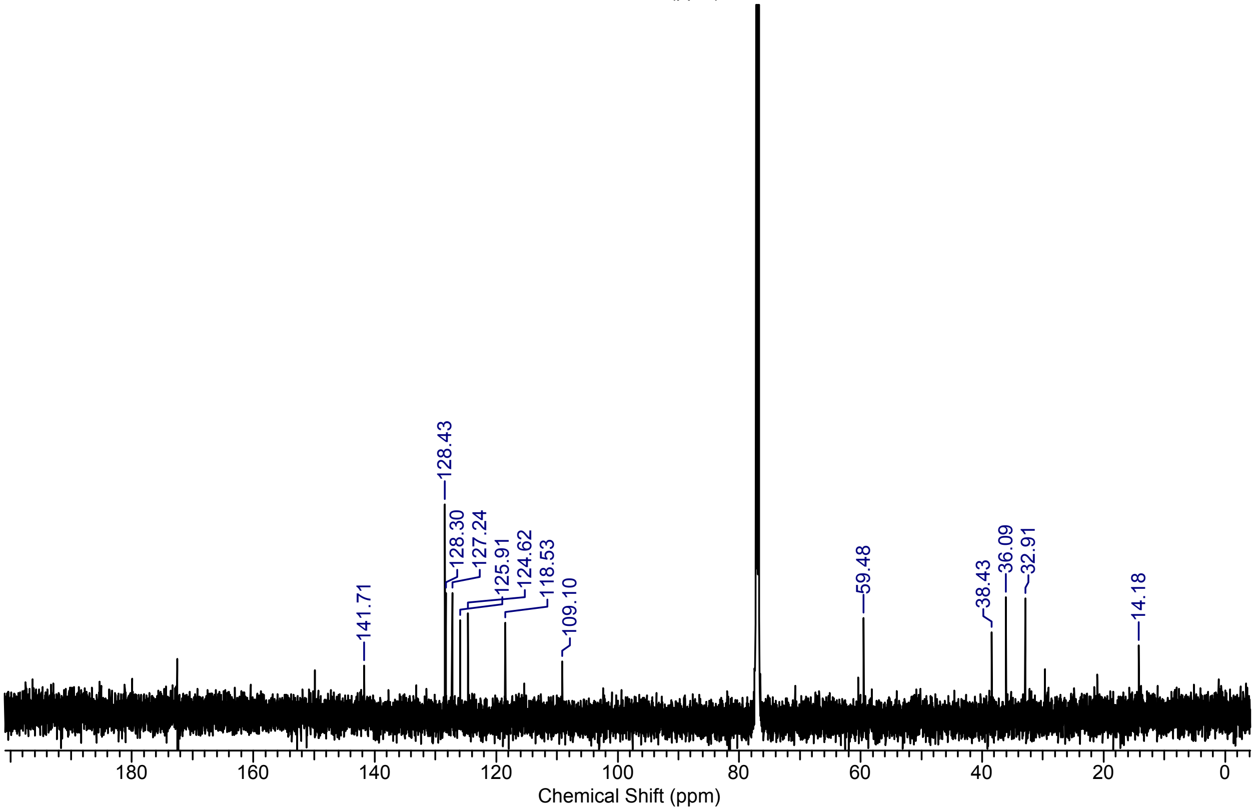

2-(pyridin-3-yl)indoline

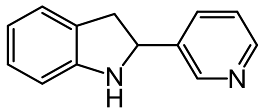

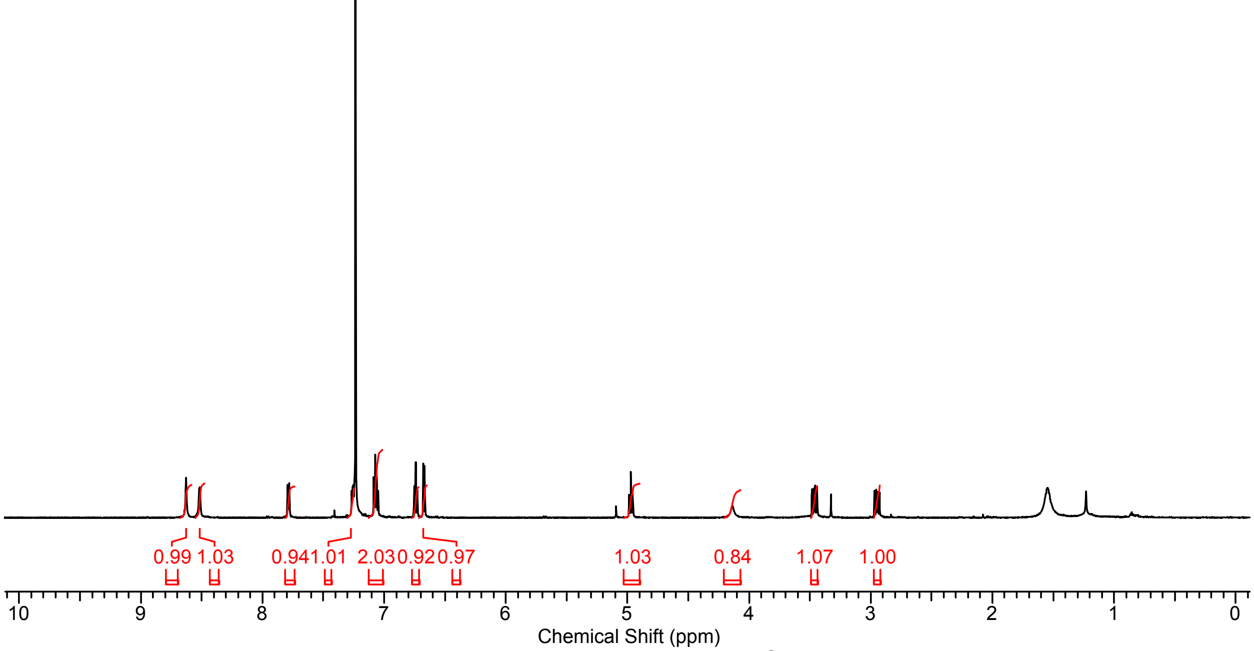

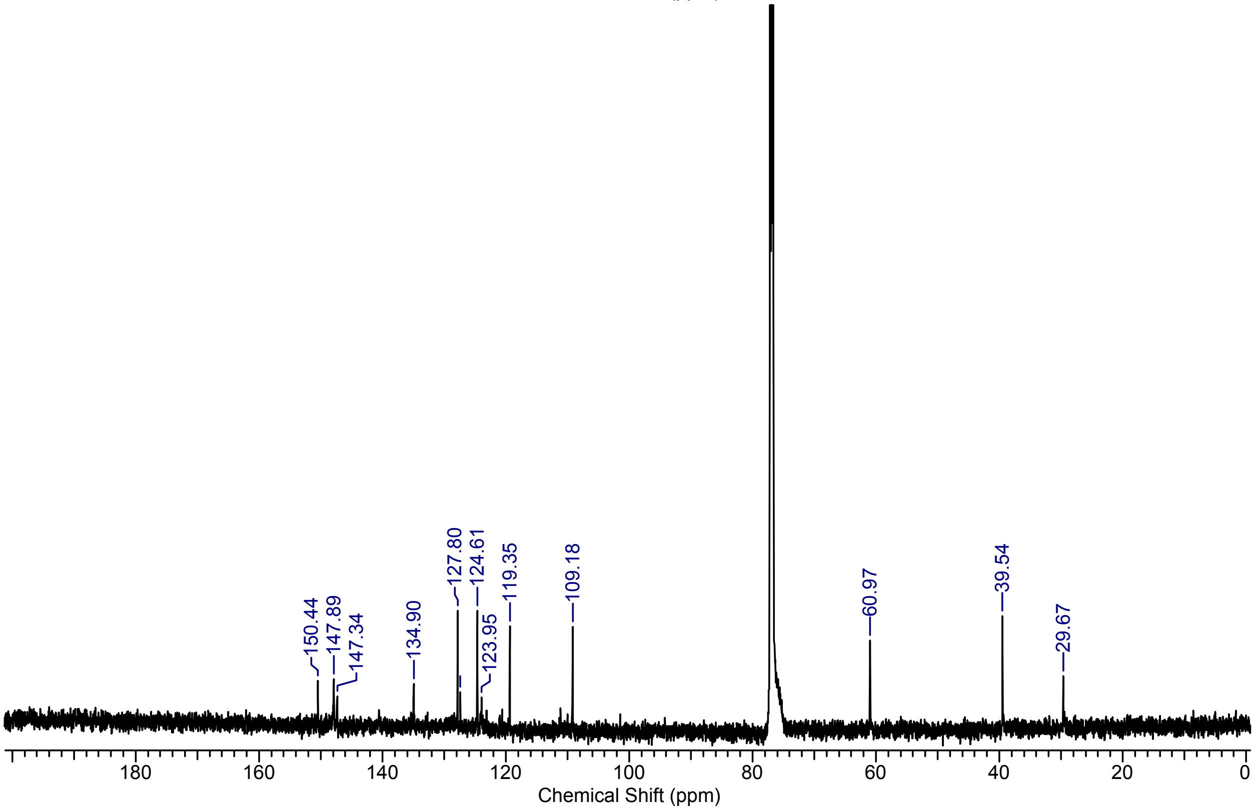

3-(indolin-2-yl)-1-(phenylsulfonyl)-1H-indole

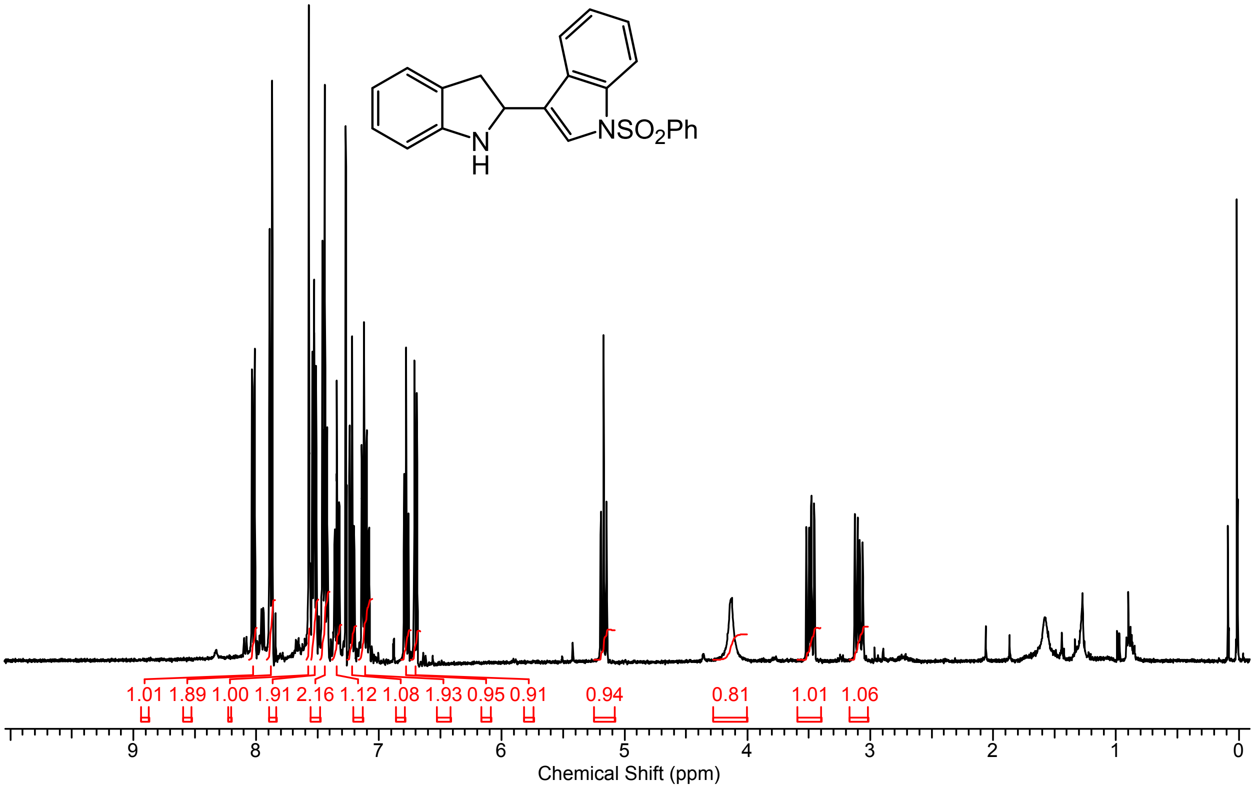

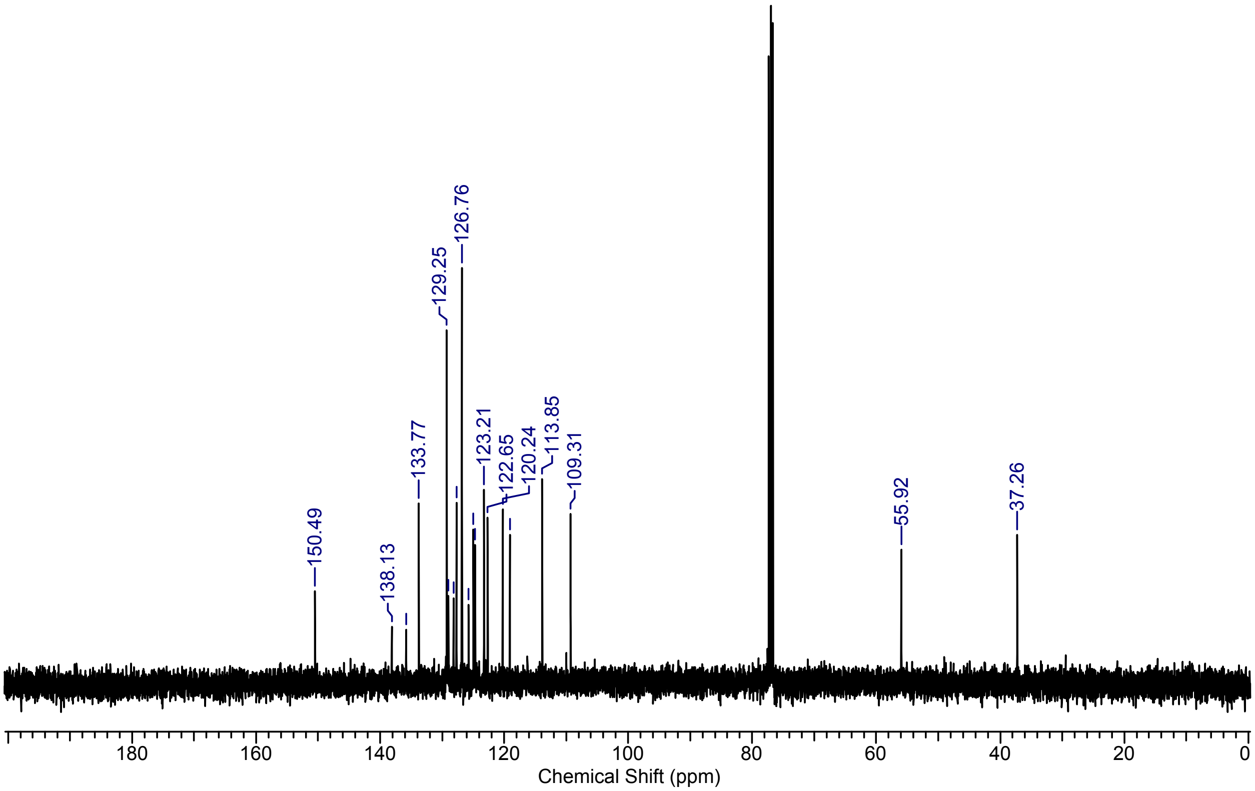

2-methyl-2-phenyl-1,2,3,4-tetrahydroquinoline

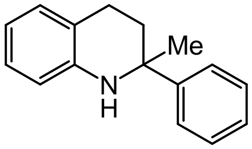

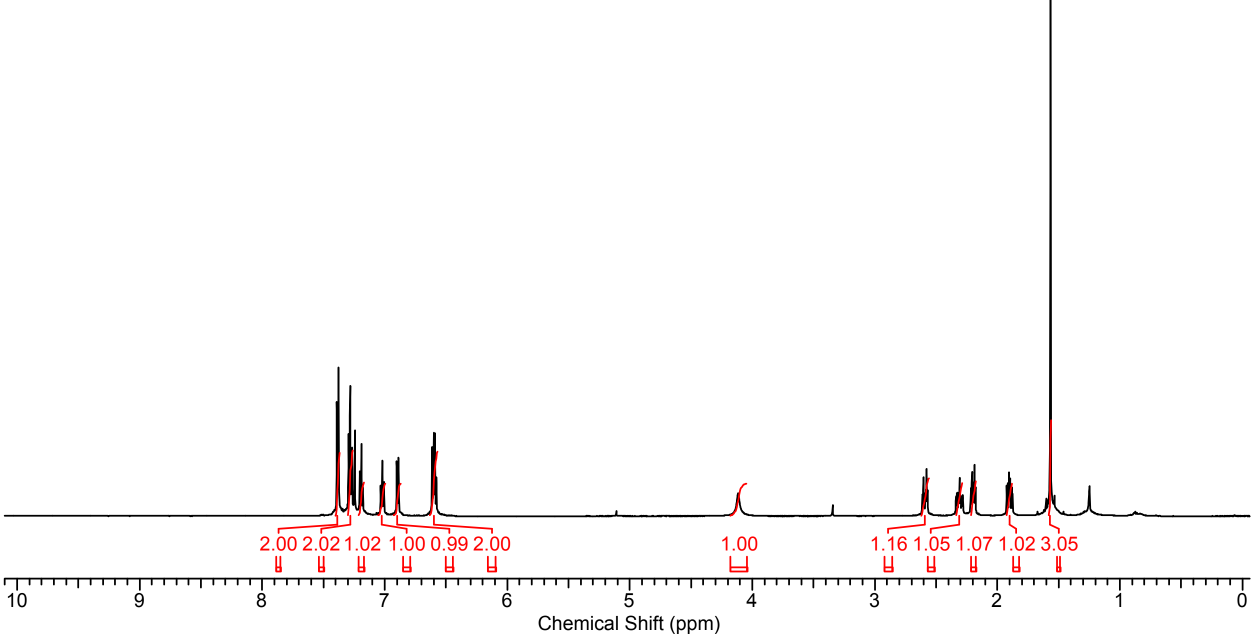

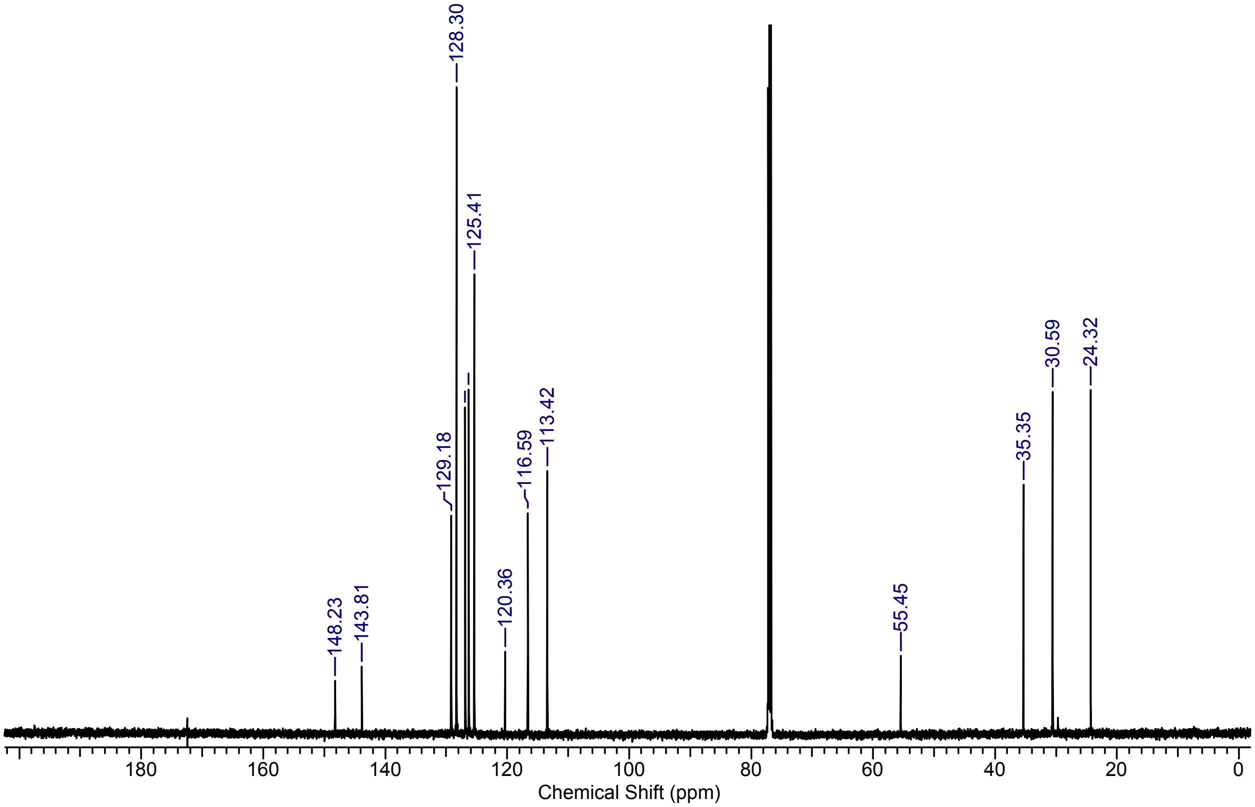

V. X-ray Crystallographic Data for Cobalt Complexes

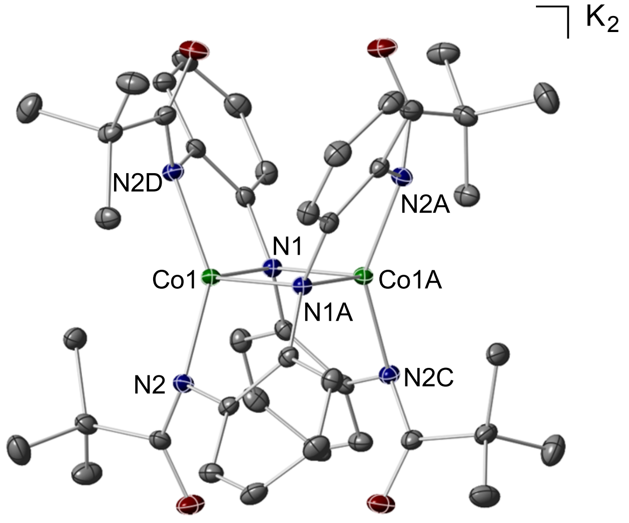

Figure S-3. Thermal ellipsoid diagram of **2** drawn at 40% probability. Hydrogen atoms and potassium counter cations have been removed for clarity. Selected bond distances (Å): Co1···Co1A 2.6586(7), Co1–N1 2.1123(16), Co–N1A 2.1123(16), Co–N2D 1.9639(14), Co1–N2 1.9639(14) and angles (°): N1–Co1–N1A 102.00(7), N1–Co–N2D 86.15(4), N1–Co1–N2 115.35(4), N2D–Co–N2 146.66(8).

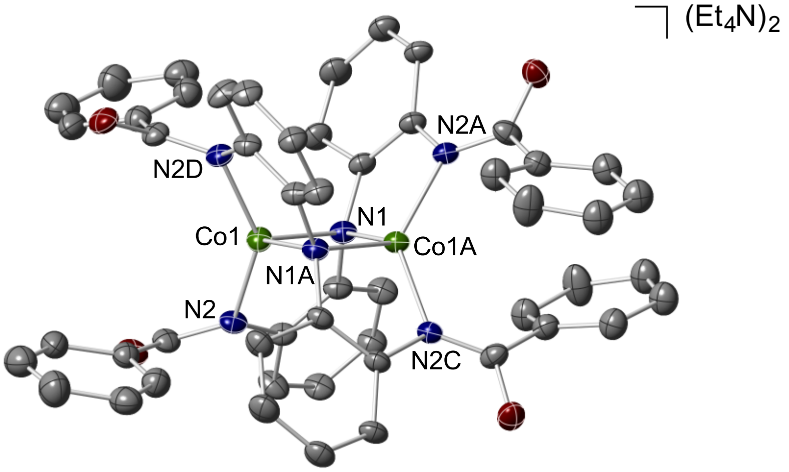

Figure S-4. Thermal ellipsoid diagram of **3** drawn at 40% probability. Hydrogen atoms and tetraethylammonium counter cations have been removed for clarity. Selected bond distances (Å): Selected bond distances (Å): Co1···Co1A 2.666(4), Co1–N1 2.0407(10), Co–N1A 2.048(10), Co–N2D 1.963(9), Co1–N2 1.963(9) and angles (°): N1–Co1–N1A 97.0(6), N1–Co–N2D 120.8(3), N1–Co1–N2 85.5(4), N2D–Co–N2 141.8(6).

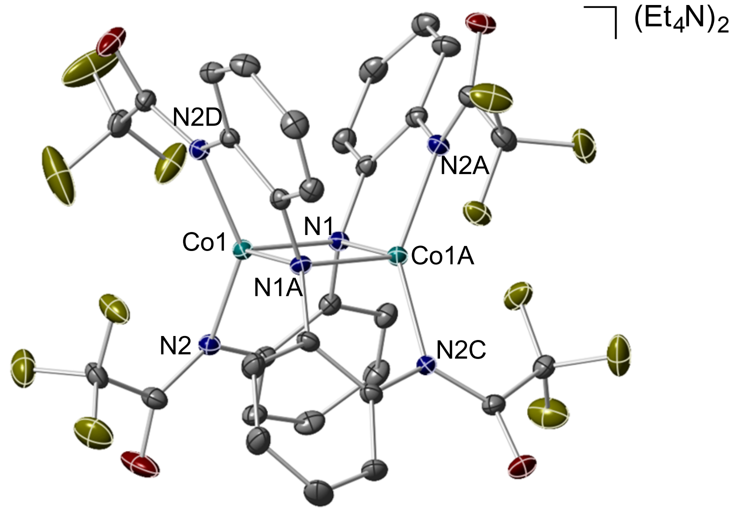

Figure S-5. Thermal ellipsoid diagram of **4** drawn at 40% probability. Hydrogen atoms and tetraethylammonium counter cations have been removed for clarity. Selected bond distances (Å): Co1···Co1A 2.631(6), Co1–N1 2.043(3), Co–N1A 2.038(3), Co–N2D 1.982(3), Co1–N2 1.986(3) and angles (°): N1–Co1–N1A 100.12(11), N1–Co–N2D 118.08(11), N1–Co1–N2 86.82(11), N2D–Co–N2 141.55(2).

Table S-2. Structural refinement data for dinuclear cobalt(II) complexes $K_2[Co_2(L^{tBu})_2]$ (**2**), $(Et_4N)_2[Co_2(L^{Ph})_2]$ (**3**) and $(Et_4N)_2[Co_2(L^{CF3})_2]$ (**4**).

	**2**	**3**	**4**
**Formula**	$C_{44}H_{52}N_6O_4K_2Co_2$	$C_{70}H_{79}N_9O_4Co_2$	$C_{52}H_{66}Co_2F_{12}N_8O_5$
**Form. Wt. (g/mol)**	924.98	1228.28	1228.99
**T (K)**	293(2)	293(2)	293(2)
**Crystal system**	Orthorhombic	Trigonal	Monoclinic
**Space group**	Fddd	P3221	P21/n
a **(Å)**	10.8136(3)	14.064(2)	10.072(2)
b **(Å)**	27.5330(7)	14.064(2)	38.683(8)
c **(Å)**	29.5266(8)	27.143(5)	14.706(3)
**α (°)**	90	90	90
**β (°)**	90	90	98.28(3)
**γ (°)**	90	120	90
**V (Å^3)**	8791.0(4)	4649(2)	5670.2(19)
**Z**	8	3	4
**ρ, calcd (g/cm^3)**	1.398	1.316	1.4396
**m/mm^{-1}**	7.994	0.59	0.677
**Crystal size (mm^3)**	0.27 × 0.13 × 0.12	0.29 × 0.15 × 0.14	0.37 × 0.13 × 0.12
**Theta range**	8.78 to 135.82	3.34 to 54.26°	3.5 to 57.4°
**Reflns. Collected**	15480	20628	101428
**Unique reflns.**	1971	4466	14606
**Data/rest./par.**	1971/0/133	4466 /444/487	14606/0/722
GOF	1.028	1.052	1.02
**Final R indexes [I>2σ (I)]**	R1 = 0.0288	R1 = 0.0814	R1 = 0.0609
**All data**	wR2 = 0.0811	wR2 = 0.1305	wR2 = 0.1705

VI. References

1. S. K. Sharma, P. S. May, M. B. Jones, S. Lense, K. I. Hardcastle and C. E. MacBeth, *Chem. Commun.*, 2011, **47**, 1827.
2. K. Sun, R. Sachwani, K. J. Richert and T. G. Driver, *Org. Lett.*, 2009, **11**, 3598.
3. S. Rousseaux, J. García-Fortanet, M. A. Del Aguila Sanchez and S. L. Buchwald, *J. Am. Chem. Soc.,* 2011, **133**, 9282.
4. G. Henrion, T. E. J. Chavas, X. Le Goff and F. Gagosz, *Angew.Chem. Int. Ed.*, 2013, **52**, 6277.
5. G. Bartoli, G. Palmieri and M. Petrini, *Tetrahedron*, 1990, **46**, 1379.
6. Z.-Y. Han, H. Xiao, X.-H. Chen and L.-Z. Gong, *J. Am. Chem. Soc.*, 2009, **131**, 9182.
7. Q. Nguyen, K. Sun and T. G. Driver, *Journal of the American Chemical Society*, 2012, **134**, 7262.
